# Network Pharmacology-Based Prediction and Verification of the Potential Mechanisms of He's Yangchao Formula against Diminished Ovarian Reserve

**DOI:** 10.1155/2022/8361808

**Published:** 2022-06-06

**Authors:** Liuqing Yang, Ying Zhao, Hongbin Xu, Yang Ma, Lin Wang, Jing Ma, Qin Zhang

**Affiliations:** ^1^Department of TCM Gynecology, Hangzhou TCM Hospital, Zhejiang Chinese Medical University, Hangzhou 310007, China; ^2^Zhejiang Chinese Medical University, Hangzhou 310053, China; ^3^Shanghai University of Traditional Chinese Medicine, Shanghai 201203, China

## Abstract

**Background:**

He's Yangchao formula (HSYC) has been clinically proven to be effective in treating diminished ovarian reserve (DOR). However, the underlying molecular mechanisms of HSYC in DOR are unclear.

**Objective:**

This study aims to predict the underlying mechanisms of He's Yangchao formula (HSYC) against DOR through network pharmacology strategies and verify *in vivo*.

**Methods:**

Systematic network pharmacology was used to speculate the bioactive components, potential targets, and the underlying mechanism of HSYC in the treatment of DOR. Then, the CTX-induced DOR mouse model was established to verify the effect of HSYC against DOR and the possible molecular mechanisms as predicted in the network pharmacology approach.

**Results:**

A total of 44 active components and 423 potential targets were obtained in HSYC. In addition, 91 targets of DOR were also screened. The identified hub genes were AKT1, ESR1, IL6, and P53. Further molecular docking showed that the four hub targets were well-bound with their corresponding compounds. *In vivo* experiments showed that HSYC could promote the recovery of the estrous cycle and increase the number of primordial, growing follicles and corpora lutea. Besides, The results of qRT-PCR showed HSYC could regulate the expression of AKT1, ESR1, P53, and IL6 in DOR mice.

**Conclusion:**

It was demonstrated that HSYC could increase ovarian reserves, and AKT1, ESR1, IL6, and P53 may play an essential role in this effect, which provided a new reference for the current lack of active interventions of DOR.

## 1. Introduction

Diminished ovarian reserve (DOR) defines as a decrease in the quantity and quantity of oocytes, which is a major cause of female infertility and assisted reproductive technology (ART) failure [1–3]. Its occurrence rate has shown rapid growth in recent years. The prevalence has risen from 19% to 26% over the past decades, representing a great challenge in modern infertility management [4, 5]. As a complex disease, DOR seriously affects the mental and physical health of women. Currently, the main pathogen of DOR is unclear. It has been reported that factors such as genetic predisposition, autoimmunity, oncologic treatments, cigarette smoking, ovarian surgery, and psychological stress are all related to the onset of DOR [6–8].

The decline of ovarian reserve is a continuous and gradual process. The premature depletion of ovarian reserve eventually results in premature ovarian insufficiency, which is a more severe condition that makes it much harder to treat. Therefore, rapid treatment is crucial for DOR women. Various treatment strategies are being used to fight against DOR, including estrogen supplementation, dehydroepiandrosterone (DHEA), ovulation induction therapy, ART, and ovum donation. DHEA is an endogenous steroid that originates from the zona reticularis of the adrenal cortex and the ovarian theca cells in women. It is produced by the conversion of cholesterol, acting an important role in the formation of estradiol in peripheral tissues [9]. DHEA is often used in the treatment of DOR. However, long-term use of DHEA can lead to some side effects [10]. Therefore, it is an urgent issue to develop a more economical, safe, and comprehensive treatment for DOR.

Traditional Medicine has been included in the influential global medical compendium by the World Health Organization. Traditional Chinese Medicine (TCM) is a branch of Traditional Medicine and is an essential element of China's national health policies. It exerts beneficial therapeutic efficacy and reduces side effects through various herbal combinations, thereby preventing and treating various diseases [11]. As shown in Table 1, He's Yangchao formula (HSYC) comprises eight-herbal medicines. It is a particular herbal prescription for DOR that originates from the ancestral experience of “*He*'*s TCM Gynecology,*” which is the famous school doctrine of Gynecology of TCM in China and has lasted for more than 170 years [12]. HSYC effectively improved the clinical symptoms, increased the AMH level and the antral follicle count, and reduced serum follicle-stimulating hormone (FSH) in DOR patients [13]. However, the mechanism of HSYC in the treatment of DOR remains unclear.

**Figure 1:**
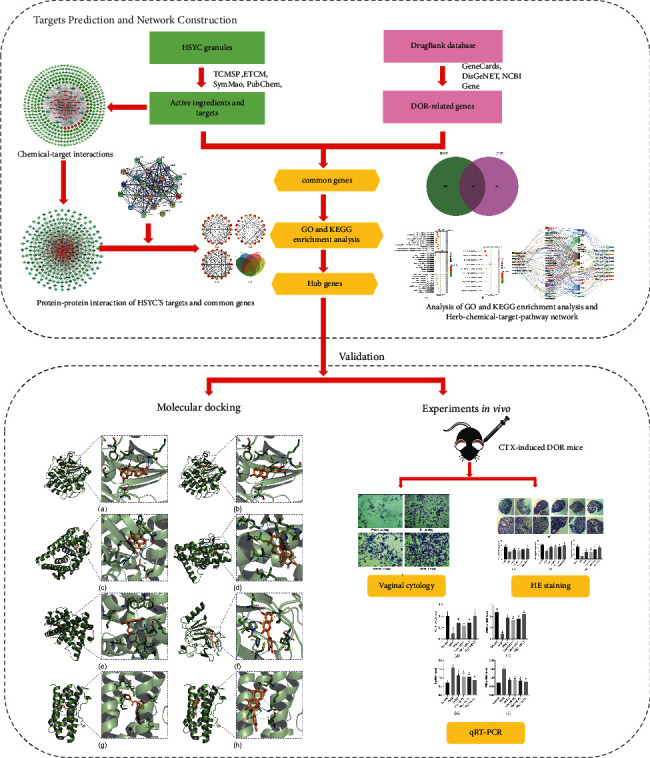
A workflow of this study, including network pharmacology prediction and validation.

Compared with western medicine, TCM is characterized by multicomponent and multitarget and exerts its effect on multiple biological processes to treat diseases via its diverse bioactive components, which act on multiple targets [14]. Network pharmacology's emergence provides us with new ideas and methods to explore the complex network relationship between multicomponent and multitarget [15]. Network pharmacology is a branch of pharmacology, which takes the theory of systems biology and multiple pharmacology as a foundation and biomolecular network as the main means of identifying the active compounds in herbal medicines and potential therapeutic targets [16]. This study identified the bioactive compounds, potential targets, and the underlying mechanism of HSYC against DOR. In addition, *in vivo* experiments were performed to validate the molecular mechanism.  Figure 1 shows the workflow of this study. This study would provide novel insights into the mechanisms underlying TCM-mediated efficacy in DOR and provide the theoretical basis for further experimental investigations.

## 2. Material and Methods

### 2.1. Screening and Obtaining the Bioactive Compounds and Potential Targets of HSYC

All bioactive compounds of 8 herbs in HSYC (*Asparagi Radix*, *Radix Puerariae*, *Angelicae Sinensis Radix*, *Platycladi Semen*, *Cuscutae Semen*, *Cistanches Herba*, *Rubi Fructus*, and *Paeoniae Radix Alba*) were obtained from Traditional Chinese Medicine Systems Pharmacology Database and Analysis Platform (TCMSP, https://ibts.hkbu.edu.hk/LSP/tcmsp.php (accessed on 15 March 2020)). TCMSP is a complete library of Chinese herbal ingredients containing more than 36,000 chemical molecules, ingredients-related targets, and their relationships with diseases [11]. TCM is mostly oral preparation and exerts various biological effects by reaching organs and tissue after absorption, distribution, metabolism, and excretion (ADME) *in vivo*, which is called the pharmacokinetics of TCM. Oral bioavailability (OB) and drug-like (DL) are the important parameters of ADME [17, 18]. The components with OB ≥ 30% and DL ≥ 0.18 were selected as candidate main bioactive components. TCMSP, the Encyclopedia of Traditional Chinese Medicine (ETCM, https://www.tcmip.cn/ETCM/index.php/Home/Index), SymMap (https://www.symmap.org/ (accessed on 15 March 2020)), and PubChem (https://pubchem.ncbi.nlm.nih.gov (accessed on 15 March 2020)) were used to retrieve and predict the potential targets of bioactive components in HSYC. The obtained potential targets, including the name, gene ID, and organism, were normalized based on the UniProt protein database (https://www.uniprot.org (accessed on 18 March 2020)).

### 2.2. Identification of DOR-Related Targets

The DOR-related targets were collected from three databases. GeneCards (https://www.genecards.org) is a database for collecting a comprehensive compendium of human genes. The keyword “diminished ovarian reserve” was used to search GeneCards database (accessed on 18 March 2020). Next, “diminished ovarian reserve” was used as a keyword to search DisGeNET (https://www.disgenet.org (accessed on 18 March 2020)). In addition, we searched the National Centre for Biotechnology Information Gene (NCBI Gene, https://www.ncbi.nlm.nih.gov/gene (accessed on 18 March 2020)).

### 2.3. Network Construction

Based on the above analyses, a visual interaction network based on the herbs-compounds-targets interactions was established by Cytoscape software. Meanwhile, to show the mechanism of action of HSYC, the protein-protein interaction (PPI) network of potential targets of the bioactive compounds in HSYC was constructed by the STRING database (https://string-db.org (accessed on 18 March 2020)) with the cut-off criterion of combined score > 0.9 and the species “*Homo sapiens*.” In addition, the targets of the active compounds in HSYC were matched with the DOR targets to obtain the intersected genes. Then, the intersected genes of compounds-DOR were submitted to the STRING database to structure another PPI network.

### 2.4. Enrichment Analysis

To illustrate the role of the potential targets in gene functional classification, functional annotation, and enriched signal pathways, Gene Ontology (GO) and Kyoto Encyclopedia of Genes and Genomes (KEGG) pathways enrichment analysis and annotation were performed using the R package “clusterProfiler” to characterize the pathways according to the intersected genes of bioactive components and DOR. GO terms with Bonferroni value <0.05 and KEGG pathways with *p* value <0.05 were considered significant.

### 2.5. The Construction of Herb-Component-Target-Pathway Interaction Network

Then, a visual herb-component-target-pathway interaction network based on interactions between the herbs, components, DOR-related targets, and pathways was established by Cytoscape software to holistically explain the mechanism of HSYC in the treatment of DOR.

### 2.6. Screening of Hub Targets and Molecular Docking Simulation

The intersected genes of the three subnetworks based on three topology parameters, including degree, betweenness, and closeness, were intersected with the 22 potential therapeutic targets to get hub genes. The crystal structures of protein receptors of hub targets were obtained from RCSB Protein Data Bank (https://www.rcsb.org/(accessed on 29 March 2020)). PyMol 2.4.0 was used to remove the original ligand, solvent molecules, and redundant protein chains and add polar hydrogen for the crystal structures of protein receptors. Then AutoDock Tools 1.5.6 was used to compute Gasteiger charges and determine the center and size of the docking box for further docking. The 3D structure of the bioactive compounds was obtained from the PubChem database. The structure was assigned MMFF94 force field and charges, made energy minimization and added polar hydrogen. AutoDock Vina, a freely available open-source program for molecular docking, was used to evaluate the binding of the bioactive compounds and their corresponding targets. Before molecular docking, all the protein structures and the bioactive compounds were converted to PDBQT format for further docking using AutoDock Tools 1.5.6. Then the bioactive compounds were docked into their corresponding proteins using AutoDock Vina. Finally, the binding affinity was calculated by AutoDock Vina and visualized the docking result by the open-source version PyMol 2.4.0 software.

### 2.7. The Construction of DOR Models

Thirty-six female C57BL/6 mice (8 weeks old) were purchased from Shanghai SIPPR-Bk Lab Animal Company, with the license number SCXK (Shanghai) 2018–0006. All animal experimental procedures were approved by the Institutional Animal Care and Use Committee of the Zhejiang Chinese Medical University (Approval number: IACUC-20180402-02). The mice were maintained under standard conditions (20°C–25°C, natural light cycle, 40%–60% relative humidity) with ad libitum access to rodent chow and water. After 1-week adaptation, mice were randomly divided into six groups (*n* = 6/group): control, DOR, low (Low-HSYC), moderate (Mod-HSYC), high (High-HSYC), and DHEA. A DOR mice model induced by cyclophosphamide (CTX) was established based on the previous study [19]. All mice in the DOR, HSYC, and DHEA groups received an intraperitoneal (ip) injection once of 90 mg/kg CTX (Sigma-Aldrich, Germany), while mice in the control group received an equal volume of saline. The DOR mice in HSYC groups received 5.88 mg/kg, 11.75 mg/kg, and 23.5 mg/kg body weight HSYC via intragastric administration daily. The DOR mice in the DHEA group were treated with 9.75 mg/kg body weight DHEA (Sigma-Aldrich, Germany) via intragastric administration daily. The mouse administration dose was calculated by referring to *Experimental Methodology of Pharmacology* compiled by Wei et al. [20]. The mice in the control and DOR groups received an equal volume of saline. It takes approximately three weeks for primordial follicles in mice to pass through the primary, secondary, preantral, and preovulatory stages [21]. Therefore, the administration should take more than three weeks to evaluate the short effects. On the 28th day, all animals were sacrificed by cervical dislocation by a skilled expert to minimize suffering. Cervical dislocation is a standard method for euthanasia of experimental mice (weight < 200 g) [22]. The blood samples and ovarian tissues were collected from each animal.

### 2.8. Evaluation of Estrous Cycle by Vaginal Cytology

Vaginal smears were obtained daily at 09 : 00 am to observe the estrus cycle. Vaginal smear samples were stained with toluidine blue (Qiagen) and analyzed under a light microscope (Olympus, Tokyo, Japan). The estrous cycle was determined according to the cytological appearance as previously reported [23].

### 2.9. Follicular Counts

The ovarian tissues were fixed in paraformaldehyde, embedded in paraffin, and then cut into 5 *μ*m thick sections. The number of follicles was observed by hematoxylin and eosin (HE) staining according to the methods previously described [24]. All HE sections were examined by two researchers (L.Q.Y. and Y.Z.).

### 2.10. Quantitative Real-Time PCR (qRT-PCR) Analysis

Total RNA was extracted from ovarian tissues using TRIzol reagent (Invitrogen, Shanghai, China). The first-strand cDNA was reversely transcribed using a Bestar qPCR RT Kit (DBI Bioscience, Ludwigshafen, Germany). 1 *µ*g of DNA sample was used for the PCR amplification of related genes according to the reaction conditions as follows: 95°C for 4 min, 38 cycles of 94°C 30 s, 60°C 30 s, 72°C 30 s. Primers were synthesized by Sangon (Shanghai, China) and listed in Table S1. A DBI Bestar® SybrGreen qPCR Master Mix kit (DBI Bioscience) and Agilent Stratagene Mx3000P Real-time PCR instrument (DBI Bioscience) were used for the amplification. The relative expression level of each gene was calculated using the 2^−ΔΔCt^ method. *β*-Actin was used as the internal reference gene.

### 2.11. Statistical Analysis

Animal data were expressed as the mean ± standard deviation (SD) from 6 mice. Statistical analysis was conducted in GraphPad Prism 6.0. Differences between groups were analyzed using an unpaired *t*-test, and differences across six groups were analyzed using one-way ANOVA. A significant level was set at *P* < 0.05.

## 3. Results

### 3.1. Herb-Compound-Target Network

**Figure 2:**
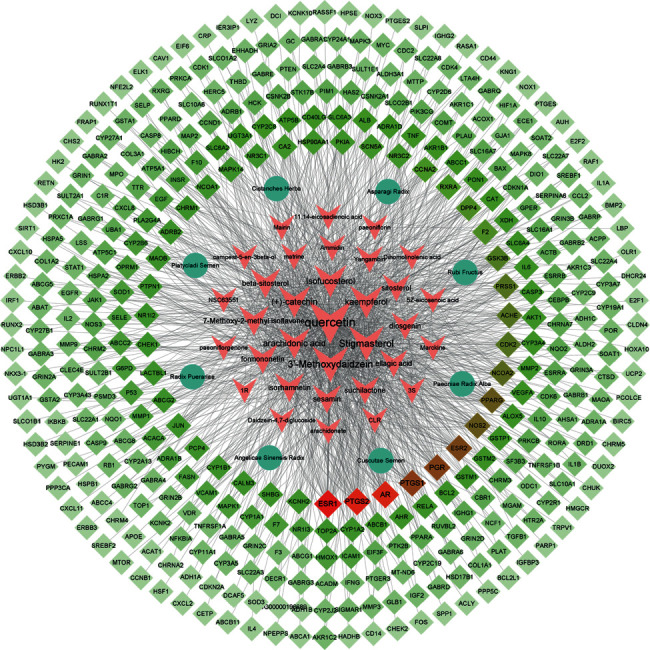
Herb-compound-target network. The size of the node represents the size of the degree value. The circles in the network represent the eight herbs in HSYC, the arrows represent the active ingredients of eight herbs, the diamonds represent the targets of the active ingredients, and the color of the nodes is illustrated from red to cyan in descending order of degree values: [3S = (3S, 5R, 8R, 9R, 10S, 14S)-3, 17-dihydroxy-4, 4, 8, 10, 14-pentamethyl-2, 3, 5, 6, 7, 9-hexahydro-1H-cyclopenta[a]phenanthrene-15,16-dione], [1R = (1R, 2R, 4aS, 6aS, 6aR, 6bR, 8aR, 12aR, 14bS)-1, 11-dihydroxy-1, 2, 6a, 6b, 9, 9, 12a-heptamethyl-10-oxo-3, 4, 5, 6, 6a, 7, 8, 8a, 13, 14b-decahydro-2H-picene-4a-carboxylic acid].

A total of 44 kinds of bioactive components in HSYC with good ADME properties were obtained. The detailed information on bioactive compounds is described in Table S2. Moreover, there were 423 targets of bioactive compounds obtained from several databases (Table S3). Then, the herb-compound-target network was constructed with 411nodes and 1429 edges (see Figure 2). In the visual interaction network, the size of the node represents the size of the degree value. The color of the nodes is illustrated from red to cyan in descending order of degree values. It can be seen from Figure 2 that quercetin, 3′-methoxydaidzein, and stigmasterol compounds, as well as ESR1, PTGS2, AR, PTGS1, and PGR genes, play a crucial role in the network.

### 3.2. PPI Network of the HSYC's Targets

**Figure 3:**
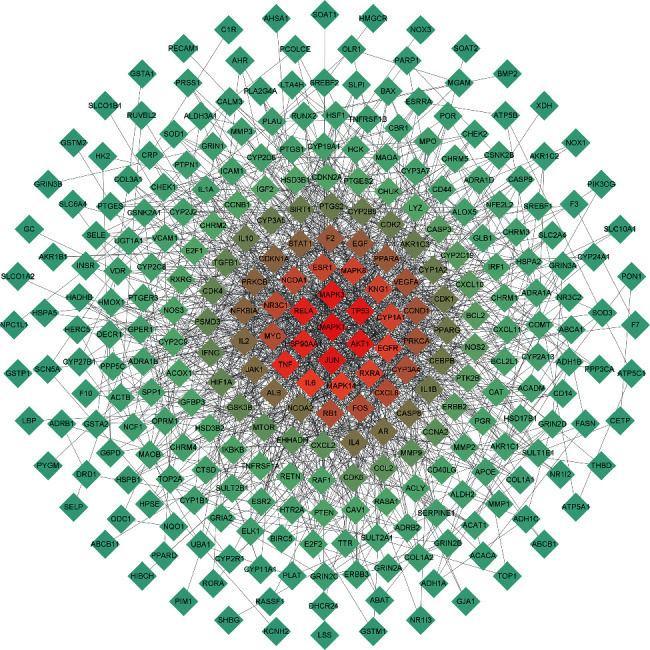
The PPI network of the HSYC's targets. The color of the nodes is illustrated from red to cyan in descending order of degree values.

The PPI network of targets in HSYC with the highest confidence was constructed using the STRING database, including 288 nodes and 1395 edges (see Figure 3). The color of the nodes is illustrated from red to cyan in descending order of degree values. So MAPK1, P53, AKTI, MAPK3, JUN RELA, HSP90AA1, TNF, and IL6 may be the main targets of biological networks regulated by HSYC.

### 3.3. PPI Network of the Potential Therapeutic Targets

**Figure 4:**
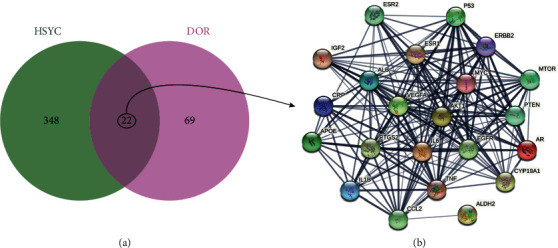
Venn Diagram and PPI network of intersected targets of HSYC and DOR. (a) Venn Diagram of intersected targets of HSYC and DOR; (b) PPI network the potential therapeutic targets.

A total of 91 DOR-related targets were obtained from the human genomic database. The number of DOR-related targets in GeneCards, DisGeNET, and NCBI Gene was 44, 43, and 28, respectively (Table S4). Twenty-two common HSYC and DOR targets (potential therapeutic targets) were obtained using the Venn Diagram tool (Figures 4(a) and 4(b)), showing the PPI network of the potential therapeutic targets.

### 3.4. KEGG Pathway Analysis and GO Functional Enrichment

**Figure 5:**
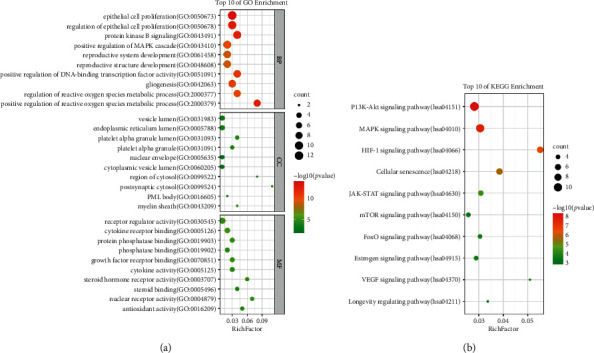
GO and KEGG enrichment analysis. The top ten significantly enriched terms in GO (biological process (BP), cellular component (CC), and molecular function (MF)) and KEGG enrichment analysis. The *Y*-axis represents the term, and the *X*-axis represents the enrichment factor. The bubble size represents the number of genes belonging to the term. The bubble color represents the value of *p* value.

The intersected targets of HSYC and DOR were analyzed by the *R* package. KEGG pathway analysis revealed that the targets of the PPI network were assigned to 89 KEGG pathways based on *P* < 0.01. GO annotation showed that the targets of the PPI network were classified into 1139 biological process (BP) terms, 20 cellular component (CC) terms, and 43 molecular function (MF) terms according to *P* < 0.01 (see Figure 5).

As shown in Figure 5(a), the targets of the PPI network in the BP ontology are primarily associated with epithelial cell proliferation, regulation of epithelial cell proliferation, positive regulation of MAPK cascade, protein kinase B signaling, reproductive structure development, reproductive system development, positive regulation of DNA-binding transcription factor activity, gliogenesis, positive regulation of reactive oxygen species metabolic process, and regulation of reactive oxygen species metabolic process. Based on GO annotation of MF, it could be seen that the targets were mainly involved in receptor regulator activity, cytokine receptor binding, growth factor receptor binding, protein phosphatase binding, cytokine activity, steroid hormone receptor activity, antioxidant activity, steroid binding, and nuclear receptor activity. For the CC ontology, the targets were mainly located in the vesicle lumen, endoplasmic reticulum lumen, nuclear chromatin, cytoplasmic vesicle lumen, platelet alpha lumen, platelet alpha, PML body, postsynaptic cytosol, region of cytosol, and the myelin sheath.

As shown in Figure 5(b), KEGG pathway enrichment analysis suggested that the targets were mainly related to signal pathways, such as the PI3K-AKT signaling pathway, MAPK signaling pathway, HIF-1 signaling pathway, ErbB signaling pathway, JAK-STAT signaling pathway, FoxO signaling pathway, estrogen signaling pathway, mTOR signaling pathway, VEGF signaling pathway, and NOD-like receptor signaling pathway. The results indicated that the targets of HSYC were distributed in different metabolic pathways. The “multicomponent, multitarget, and multipathway” mutual regulation is the possible mechanism for the treatment of DOR.

### 3.5. Screening of Hub Targets and Docking Results Analysis

**Figure 6:**
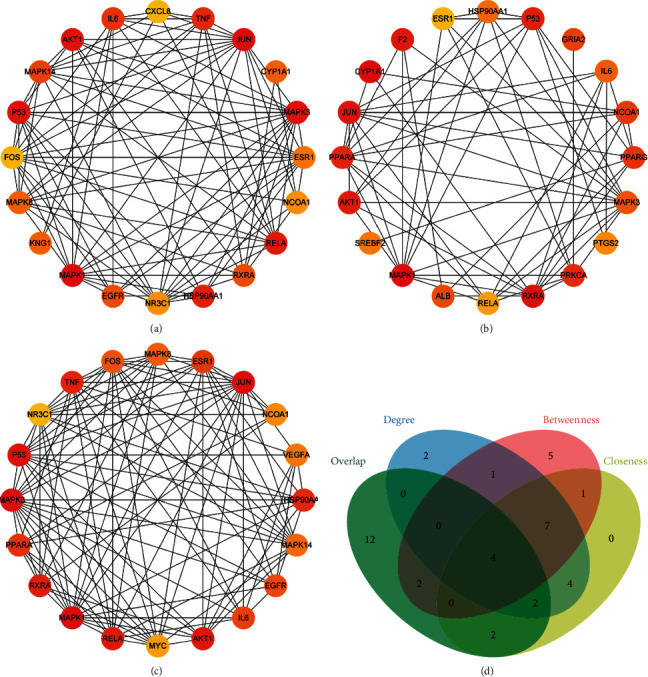
The protein-protein interaction (PPI) network of three subnetworks and hub genes screening. The three subnetworks of PPI according to degree (a), betweenness (b), and closeness (c). (d) Venn Diagram of three subnetworks and four hub targets, and the four hub targets are AKT1, ESR1, P53, and IL6.

Network analyzer, a plug-in of Cytoscape, was used to calculate the node topology parameters of the PPI network of the bioactive compounds' potential targets in HSYC. The top 20 genes screened by degree, betweenness, and closeness were used to construct the subnetworks (Figures 6(a)–6(c)). In our study, four hub targets, including AKT1, ESR1, P53, and IL6, were identified from the Venn map (Figure 6(d)).

**Figure 7:**
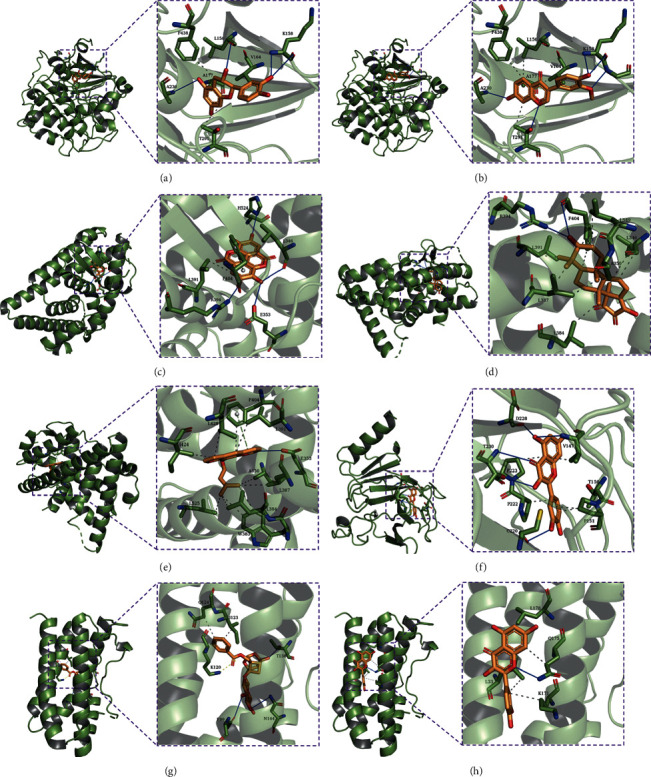
Molecular docking results. (a) The binding poses of AKT1 complexed with (+)-catechin. (b) The binding poses of AKT1 complexed with 3′-methoxydaidzein. (c) The binding poses of ESR1 complexed with ellagic acid. (d) The binding poses of ESR1 complexed with (3S, 5R, 8R, 9R, 10S, 14S)-3, 17-dihydroxy-4, 4, 8, 10, 14-pentamethyl-2, 3, 5, 6, 7, 9-hexahydro-1H-cyclopenta[a]phenanthrene-15, 16-dione. (e) The binding poses of ESR1 complexed with ammidin. (f) The binding poses of P53 complexed with quercetin. (g) The binding poses of IL6 complexed with paeoniflorin. (h) The binding poses of IL6 complexed with quercetin.

Text, the four hub targets were selected to perform molecular docking with their corresponding compounds. AutoDock Vina evaluated the small molecules' binding strength with proteins mainly through the binding energy (affinity). If the affinity is less than zero, it means that the ligand can spontaneously bind to the receptor. The smaller the value is, the easier the bioactive component is to bind to the receptor. As shown in Table 2, a total of 36 pairs of docking results were obtained. Among the docking results, most binding complexes possessed high binding affinity, which means these compounds may bind to the four hub targets to improve ovarian reserve.  Figure 7 shows the binging mode of hub targets with their corresponding compounds with high binding affinity.

### 3.6. Herb-Component-Target-Pathway Interaction Network

**Figure 8:**
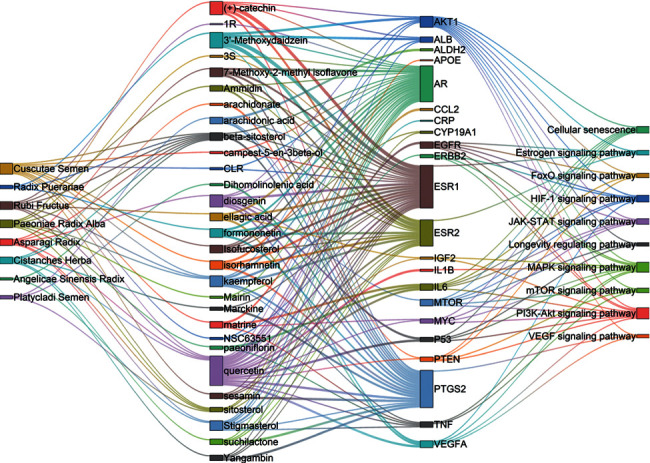
The herb-component-target-pathway interaction network. The squares represent herbs, components, targets and pathways; the edges represent the interaction of each other: [3S = (3S, 5R, 8R, 9R, 10S, 14S)-3, 17-dihydroxy-4, 4, 8, 10, 14-pentamethyl-2, 3, 5, 6, 7, 9-hexahydro-1H-cyclopenta[a]phenanthrene-15, 16-dione], [1R = (1R, 2R, 4aS, 6aS, 6aR, 6bR, 8aR, 12aR, 14bS)-1,11-dihydroxy-1, 2, 6a, 6b, 9, 9, 12a-heptamethyl-10-oxo-3, 4, 5, 6, 6a, 7, 8, 8a, 13, 14b-decahydro-2H-picene-4a-carboxylic acid].

Then, a herb-compound-target-pathway network (see Figure 8) was constructed. In the visual interaction network, the squares represent herbs, components, targets, and pathways; the edges represent the interaction of each other. In the network, the four hub genes (AKT1, ESR1, IL6, and P53) regulate the top 10 significant pathways, targeted by an average of 8.5 compounds and all the herbs.

### 3.7. HSYC Administration Increases Body Weight and Improves the Estrous Cycles of DOR Mice

**Figure 9:**
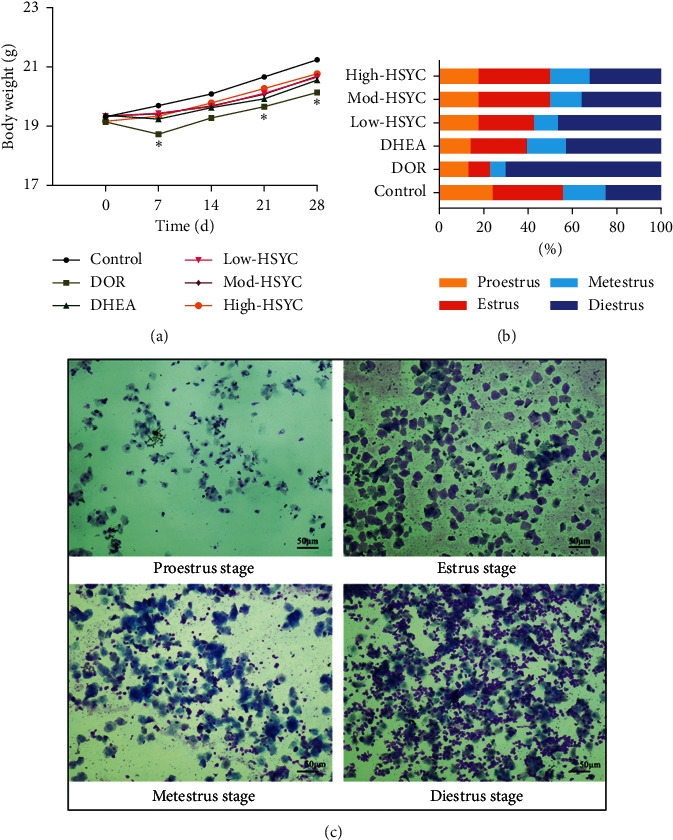
The body weight and the estrus cycle in mice. (a) Body weight changes of mice in each group. Data are expressed as mean ± SD. ^*∗*^*P* < 0.05, compared with the control group. (b) The proportion of time (days) at the four estrous cycle stages of each group within 28 days of drug treatment. (c) The representative staining of vaginal smears indicated the proestrus, estrus, metestrus, and diestrus cycle stages, respectively.

As shown in Figure 9(a), mice in the DOR groups exhibited significantly lower body weight than mice in the other groups on the 7th, 21st, and 28th day (*P* < 0.05). In addition, The mice's estrous cycle was continuously measured to evaluate the protective effects of HSYC on ovarian function. The diestrus stage of the DOR group was significantly prolonged during the 28 days of experiments, as presented in Figure 9(b). This abnormality did not completely recover to normal lengths, but it was attenuated in the DHEA and HSYC groups, with a better effect in the High-HSYC group. The normal estrous cycle has four stages, including proestrus, estrus, metestrus, and diestrus stages, as shown in Figure 9(c).

### 3.8. HSYC Improves the Ovarian Structure and Follicle Development in DOR Mice

**Figure 10:**
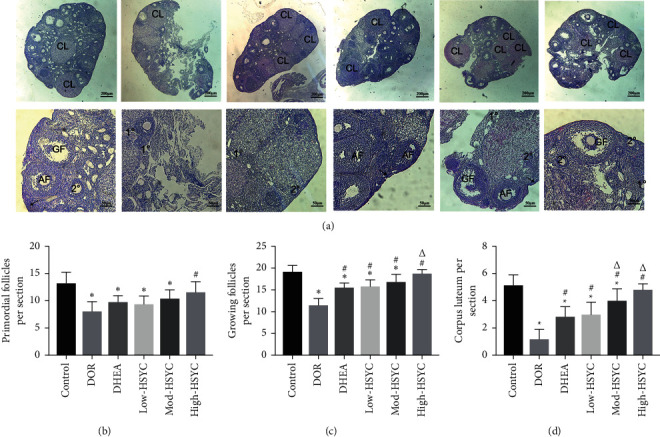
HSYC administration prevents the loss of different follicle types caused by CTX treatment. (a) Hematoxylin and eosin staining of ovaries. Arrow (⟶): primordial follicle; 1°: primary follicle; 2°: secondary follicle; AF: antral follicle; GF: mature follicle; CL: corpus luteum. (b) The number of primordial follicles. (c) The number of growing follicles. (d) The number of corpora lutea. Data are expressed as mean ± SD. ^*∗*^*P* < 0.05, compared with the control group; ^#^*P* < 0.05, compared with the DOR group; ^Δ^*P* < 0.05, compared with the DHEA group and HSYC groups.

The histological analysis of the ovaries is presented in Figures 10(a)–10(d) . Compared with the control group, except for the High-HSYC group, the numbers of primordial follicles, growing follicles (including primary follicles, secondary follicles, and anal follicles), and corpus luteum were significantly reduced in the other groups (Figure 10(a)–10(d), *P* < 0.05). This result shows that high-dose HSYC can prevent the loss of different types of follicles caused by CTX. Both HSYC and DHEA can significantly increase the number of growing follicles and the number of corpus luteum (*P* < 0.05), and the effect of HSYC is better than that of DHEA (Figures 10(a), 10(c), and 10(d), *P* < 0.05).

### 3.9. HSYC Regulates the mRNA Expressions of the Hub Targets

**Figure 11:**
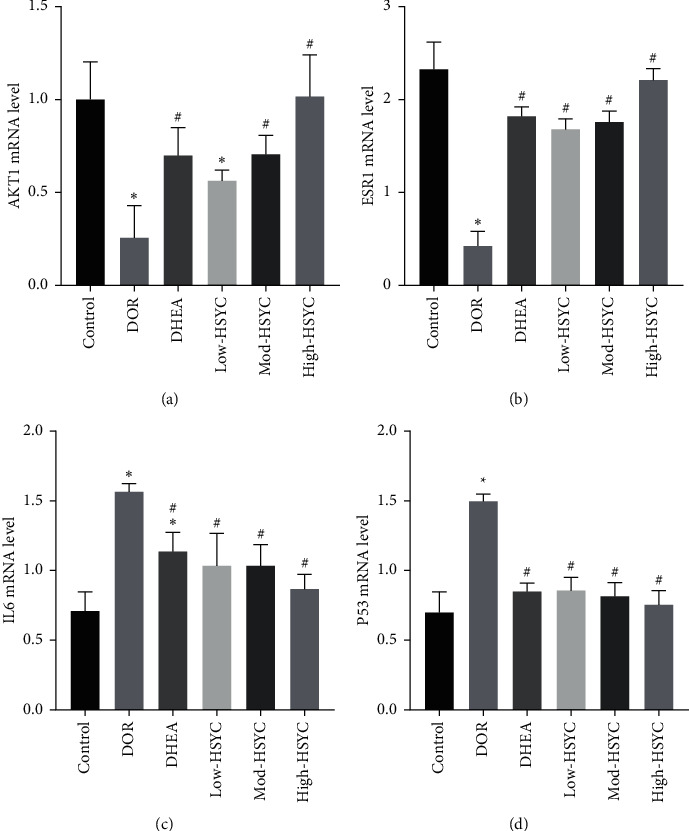
The mRNA expression of four hub targets. The relative mRNA expression levels of AKT1, ESR1, IL6, and P53 genes, respectively. Data are expressed as mean ± SD. ^*∗*^*P* < 0.05, compared with the control group; ^#^*P* < 0.05, compared with the DOR group.

In contrast to the control group, the mRNA expressions of AKT1 and ESR1 declined in the DOR group (*P* < 0.05); the expressions then elevated after HSYC and DHEA treatment (Figures 11(a) and 11(b), *P* < 0.05). In addition, the expressions of IL6 and P53 have the opposite tendency. The mRNA expressions of IL6 and P53 were upregulated in DOR mice, while HSYC and DHEA could significantly inhibit their expression levels (Figures 11(c) and 11(d), *P* < 0.05). Moreover, in inhibiting P53 mRNA expression, high-dose HSYC is more significant than DHEA (Figure 11(d). *P* < 0.05).

## 4. Discussion

Nowadays, delaying pregnancy and parenthood has become a global phenomenon, especially in high-resource countries. However, females' ovarian reserve falls sharply after the age of 35. In addition to causing reproductive dysfunction, DOR also increases cardiovascular disease and depression [25]. Clinical findings have confirmed that HSYC is effective in treating DOR, but the underlying mechanism remains unknown. The network pharmacology approach, which integrates systems biology and in silico technologies, may offer a direction for the mechanistic study of complicated TCM [16]. In the current study, we used this approach to clarify the pharmacological mechanism of HSYC on DOR.

In the system of TCM, compounds lacking proper pharmacokinetic properties could not reach the target organs to deliver the biological activities [26]. In the current study, the compounds in HSYC with OB ≥ 30% and DL ≥ 0.18 were considered pharmacokinetically active as they are possibly absorbed and distributed in the human body. In the herb-compound-target-disease network, compounds with high-degree may account for the major therapeutic effects of HSYC on DOR. In our study, there were 44 bioactive compounds in HSYC. Quercetin and *β*-sitosterol have a high degree, which suggested that both of them may have major therapeutic effects. Quercetin has extensive biological properties, including antioxidant, anti-inflammatory, and antiapoptosis [27, 28]. It has been shown that quercetin significantly increased the primordial follicle number and AMH level and decreased atretic follicle number in CTX-induced DOR mice [29, 30]. *β*-Sitosterol is another significant bioactive compound. The pharmaceutical properties of *β*-sitosterol, like anti-infection, anti-inflammatory activity, antioxidant, and estrogenic functions, have been reported [31, 32]. Mohammadrezaei et al. reported that the treatment with *β*-sitosterol increased testosterone and ethoxyresorufin-O-deethylase levels in Kutum's fertilized egg [33]. These findings might indicate the potential therapeutic efficacy of our present eight-herbal medicine in DOR.

The KEGG pathways of HSYC against DOR include the PI3K-AKT signaling pathway, MAPK signaling pathway, and HIF-1 signaling pathway. Existing studies reported that PI3K/AKT had been shown to have an important role within DOR. Studies have shown that the PI3K/AKT pathway's activation plays a crucial role in folliculogenesis, including follicle recruitment, development, and maturation [34–36]. Besides, suppressing MAPK signaling could inhibit DOR and preserve ovarian function and structure [37, 38]. HIF-1 is a master regulator of cellular adaptation to hypoxia and has been suggested as a potential therapeutic target in DOR. HIF-1 contributed to granulosa cell proliferation, which is crucial for ovarian follicle growth by regulating cell proliferation and follicle-stimulating hormone-mediated autophagy [39].

The four hub targets, AKT1, ESR1, P53, and IL6, were created as 3D graphs of docking and analysis. Further molecular docking showed that the four hub targets were well-bound with their corresponding compounds. AKT1 is the important gene in the PI3K-AKT signaling pathway. Also, AKT1 is a multifunctional protein regulating cell growth, survival, and proliferation [40]. It plays a critical role in both the growth and maturation of the oocyte. AKT1 could inhibit follicular cell apoptosis by reducing BAX and FOXO3a *in vitro* cultures [41]. AKT1−/− females mice display a reduced number of growing antral follicles and abnormal estrous cyclicity [42]. As the primary female sex hormones, estrogens exert their functions by binding to estrogen receptor *α* (ER*α*), encoded by the gene ESR1 [43, 44]. ESR1 knockout mice showed reproductive impairment as anovulation and complete infertility [45]. Several studies have reported that ESR1 polymorphism is associated with primary ovarian failure (POF) [46, 47]. Professor Zi-Jiang Chen's team conducted a genetic analysis on 371 unrelated idiopathic POF patients and 800 normal women (all of Han nationality) and found that ESR1 gene mutations are significantly related to POF [48]. Many studies have also revealed that CTX-induced loss of ovarian reserve involves the activation of P53 [49]. P53 can directly activate the transcription of various proapoptotic genes in CTX-induced ovarian damage mice [49]. Similarly, Stefania et al. used ChIP experiments to confirm that P53 plays a direct role in CTX-induced apoptosis in mouse oocytes [50]. Besides, IL6 is one of the inflammatory cytokines and plays an irreplaceable role in the inflammatory response. Recent studies have shown that inflammation is a crucial marker of the aging ovarian stroma and is considered a new DOR mechanism [51, 52]. Clinical studies in humans have shown that inflammatory marker levels are associated with the risk of DOR. Yue et al. found that compared to the control group, the serum level of IL6 in the primary ovarian failure group was significantly higher [53]. By molecular docking, we found that AKT1, ESR1, P53, and IL6 have the good binding ability with bioactive compounds in HSYC. Therefore, based on the molecular docking results and combining these hub targets' physiological roles in the ovaries, we speculate that HSYC could play a beneficial role in DOR mice via AKT1, ESR1 P53, and IL6.

Next, we studied the efficiency and mechanism of HSYC on DOR mice through animal experiments. A regular estrous cycle that lasts for 4 to 6 days in mice can be a key index of the integrity of the HPO reproductive axis [54]. In the present study, HE staining results revealed that HSYC could significantly improve the estrous cycle. These results indicate a protective role of HSYC on the DOR mice HPO axis. In addition, the primordial follicle pool is nonrenewable. Their development ends with either ovulation or atresia. Therefore, it represents the most important follicular [54]. This study showed that high-dose HSYC completely rescued the loss of primordial follicles induced by CTX and increased the total number of growing follicles and corpus luteum. These suggested that HSYC could improve ovarian reserve and have protective effects against CTX damage on the ovaries.

The results of qRT-PCR suggested that the expression of AKT1 and ESR1 was downregulated, and the expression of P53 and IL6 was upregulated in DOR mice. HSYC could promote the expression of AKT1 and ESR1 and inhibit the expression of P53 and IL6. These are consistent with previous reports. These results were consistent with the results predicted by network pharmacology and molecular docking.

As with any study, this study did have some limitations. First, due to public databases being updated in real-time, this study can only partially elucidate the molecular mechanism by which HSYC improves ovarian reserve. Second, only four hub genes were verified by molecular docking, and *in vitro* experiments, other possible pathways should be performed through *in vivo* and *in vitro* experiments in the future.

## 5. Conclusions

Taken together, HSYC may ameliorate ovarian reserve against CTX-induced DOR via the regulation of multiple targets. Our study further suggested that combined network pharmacology prediction and experimental validation study may offer a valuable tool to characterize the action mechanism of TCM.

## Figures and Tables

**Table 1:** Herbal constituents of HSYC.

Chinese name	Pinyin name	Latin name	Parts	Weight/dose (g)
天冬	Tiandong	*Asparagi Radix*	Root	10
葛根	Gegen	*Radix Puerariae*	Root	20
当归	Danggui	*Angelicae Sinensis Radix*	Root	10
柏子仁	Baiziren	*Platycladi Semen*	Ripe Kernel	10
菟丝子	Tusizi	*Cuscutae Semen*	Seed	10
肉苁蓉	Roucongrong	*Cistanches Herba*	Fleshy Stem	10
覆盆子	Fupenzi	*Rubi Fructus*	Fruit	10
白芍	Baishao	*Paeoniae Radix Alba*	Root	10

**Table 2:** Docking results of the four hub targets with their corresponding compounds.

Mol ID	Compound	Target	Affinity (kcal/mol)
MOL000492	(+)-Catechin	AKT1	−7.4
MOL000546	Diosgenin	AKT1	−2.7
MOL000422	Kaempferol	AKT1	−7.1
MOL000098	Quercetin	AKT1	−7.2
MOL002959	3′-methoxydaidzein	AKT1	−8.4
MOL000492	(+)-Catechin	ESR1	−7.3
MOL002959	3′-methoxydaidzein	ESR1	−6
MOL003896	7-Methoxy-2-Methyl Isoflavone	ESR1	−6.6
MOL001927	Albiflorin	ESR1	−6.7
MOL000546	Diosgenin	ESR1	−8.1
MOL001002	Ellagic acid	ESR1	−9.6
MOL010586	Formononetin	ESR1	−6
MOL000354	Isorhamnetin	ESR1	−6.3
MOL000422	Kaempferol	ESR1	−7.2
MOL001924	Paeoniflorin	ESR1	−7
MOL000098	Quercetin	ESR1	−7.3
MOL000449	Stigmasterol	ESR1	−6.2
MOL001919	(3S,5R,8R,9R,10S,14S)-3,17-Dihydroxy-4,4,8,10,14-pentamethyl-2,3,5,6,7,9-hexahydro-1H-cyclopenta[a]phenanthrene-15,16-dione	ESR1	−8.6
MOL001941	Ammidin	ESR1	−8.6
MOL005320	Arachidonic acid	ESR1	−7.3
MOL008583	Beta-Sitosterol	ESR1	−8.1
MOL005043	Campest-5-En-3beta-Ol	ESR1	−6.6
MOL000953	CLR	ESR1	−6.4
MOL005440	Isofucosterol	ESR1	−6.9
MOL000211	Mairin	ESR1	−7.4
MOL008871	Marckine	ESR1	−7.9
MOL000184	Nsc63551	ESR1	−6.7
MOL001558	Sesamin	ESR1	−6.2
MOL000359	Sitosterol	ESR1	−6.7
MOL005384	Suchilactone	ESR1	−6.6
MOL007563	Yangambin	ESR1	−5.6
MOL000546	Diosgenin	P53	−6.6
MOL000098	Quercetin	P53	−7.7
MOL005944	Matrine	IL6	−5.9
MOL001924	Paeoniflorin	IL6	−6.7
MOL000098	Quercetin	IL6	−6.4

## Data Availability

The data used to support the findings of this study are included within the article and the supplementary materials.

## Supplementary Materials

Supplementary Materials Table S1. The primer sequences used in this present study. Table S2. Information of bioactive compounds in HSYC with good ADME properties. Table S3. Targets of bioactive compounds obtained from databases. Table S4. DOR-related targets.
