# Supplementary material for: Network Pharmacology-Based Prediction and Verification of the Potential Mechanisms of He's Yangchao Formula against Diminished Ovarian Reserve
Source: Evid Based Complement Alternat Med. 2022 Jun 6;2022:8361808. doi: 10.1155/2022/8361808 (PMC9192314; doi:10.1155/2022/8361808)
Supplement: Supplementary Materials — Table S1. The primer sequences used in this present study. Table S2. Information of bioactive compounds in HSYC with good ADME properties. Table S3. Targets of bioactive compounds obtained from databases. Table S4. DOR-related targets. [file 8361808.f1.zip › 8361808.f1/Table S1.pdf]

Table S1. The primer sequences used in this present study.

**Gene**	**Sequence (5’-3’)**
AKT1	CCC TTC TAC AAC CAG GAC CA
	ATA CAC ATC CTG CCA CAC GA
ESR1	AAT GAA ATG GGT GCT TCA GG
	ATA GAT CAT GGG CGG TTC AG
TP53	AGA GAC CGC CGT ACA GAA GA
	CTG TAG CAT GGG CAT CCT TT
IL6	AGT TGC CTT CTT GGG ACT GA
	TCC ACG ATT TCC CAG AGA AC
β-actin	CGC AGC CAC TGT CGA GTC
	GTC ATC CAT GGC GAA CTG GT
