# Supplementary material for: Network Pharmacology-Based Prediction and Verification of the Potential Mechanisms of He's Yangchao Formula against Diminished Ovarian Reserve
Source: Evid Based Complement Alternat Med. 2022 Jun 6;2022:8361808. doi: 10.1155/2022/8361808 (PMC9192314; doi:10.1155/2022/8361808)
Supplement: Supplementary Materials — Table S1. The primer sequences used in this present study. Table S2. Information of bioactive compounds in HSYC with good ADME properties. Table S3. Targets of bioactive compounds obtained from databases. Table S4. DOR-related targets. [file 8361808.f1.zip › 8361808.f1/Table S2.pdf]

Table S2 Information of bioactive compounds in HSYC with good ADME properties

Herb	Molecule Name	OB(%)	DL
Asparagi Radix	beta-sitosterol	36.91	0.75
Asparagi Radix	sitosterol	36.91	0.75
Asparagi Radix	methylprotodioscin_qt	35.12	0.86
Asparagi Radix	pseudoprotodioscin_qt	37.93	0.87
Asparagi Radix	7-Methoxy-2-methyl isoflavone	42.56	0.2
Asparagi Radix	Asparaside A_qt	30.6	0.86
Asparagi Radix	Stigmasterol	43.83	0.76
Asparagi Radix	diosgenin	80.88	0.81
Asparagi Radix	quercetin	46.43	0.28
Radix Puerariae	formononetin	69.67	0.21
Radix Puerariae	beta-sitosterol	36.91	0.75
Radix Puerariae	3'-Methoxydaidzein	48.57	0.24
Radix Puerariae	Daidzein-4,7-diglucoside	47.27	0.67
ngelicae Sinensis Rad	beta-sitosterol	36.91	0.75
ngelicae Sinensis Rad	Stigmasterol	43.83	0.76
Platycladi Semen	arachidonic acid	45.57	0.2
Platycladi Semen	11,14-eicosadienoic acid	39.99	0.2
Platycladi Semen	sitosterol	36.91	0.75
Platycladi Semen	Dihomolinolenic acid	44.11	0.2
Platycladi Semen	5Z-eicosenoic acid	30.7	0.2
Cuscutae Semen	sesamin	56.55	0.83
Cuscutae Semen	NSC63551	39.25	0.76
Cuscutae Semen	isorhamnetin	49.6	0.31
Cuscutae Semen	beta-sitosterol	36.91	0.75
Cuscutae Semen	kaempferol	41.88	0.24
Cuscutae Semen	campest-5-en-3beta-ol	37.58	0.71
Cuscutae Semen	Isofucosterol	43.78	0.76
Cuscutae Semen	matrine	63.77	0.25
Cuscutae Semen	sophranol	55.42	0.28
Cuscutae Semen	Cuscutoside B	47.56	0.18
Cuscutae Semen	CLR	37.87	0.68
Cuscutae Semen	quercetin	46.43	0.28
Cistanches Herba	beta-sitosterol	36.91	0.75
Cistanches Herba	arachidonate	45.57	0.2
Cistanches Herba	suchilactone	57.52	0.56
Cistanches Herba	Yangambin	57.53	0.81
Cistanches Herba	quercetin	46.43	0.28
Cistanches Herba	3-tetrahydro-3H-pyrrolizin-1-yl]methyl (2R)-2-hydroxy-2-(1-hydrox	35.35	0.18
Cistanches Herba	Marckine	37.05	0.69
Rubi Fructus	ellagic acid	43.06	0.43
Rubi Fructus	Ammidin	34.55	0.22
Rubi Fructus	beta-sitosterol	36.91	0.75
Rubi Fructus	sitosterol	36.91	0.75
Rubi Fructus	kaempferol	41.88	0.24
Rubi Fructus	·dihydroxy-1,2,6a,6b,9,9,12a-heptamethyl-10-oxo-3,4,5,6,6a,7,8,8a,	51.16	0.72
Rubi Fructus	quercetin	46.43	0.28
Paeoniae Radix Alba	ıa,12alpha-epoxy-3beta-23-dihydroxy-30-norolean-20-en-28,12beta	64.77	0.38
Paeoniae Radix Alba	paeoniflorgenone	87.59	0.37
Paeoniae Radix Alba	ıydroxy-4,4,8,10,14-pentamethyl-2,3,5,6,7,9-hexahydro-1H-cyclope	43.56	0.53
Paeoniae Radix Alba	Lactiflorin	49.12	0.8
Paeoniae Radix Alba	paeoniflorin	53.87	0.79
Paeoniae Radix Alba	paeoniflorin_qt	68.18	0.4
Paeoniae Radix Alba	albiflorin_qt	66.64	0.33
Paeoniae Radix Alba	benzoyl paeoniflorin	31.27	0.75
Paeoniae Radix Alba	Mairin	55.38	0.78
Paeoniae Radix Alba	beta-sitosterol	36.91	0.75
Paeoniae Radix Alba	sitosterol	36.91	0.75
Paeoniae Radix Alba	kaempferol	41.88	0.24
Paeoniae Radix Alba	(+)-catechin	54.83	0.24
