# Supplementary material for: Network Pharmacology-Based Prediction and Verification of the Potential Mechanisms of He's Yangchao Formula against Diminished Ovarian Reserve
Source: Evid Based Complement Alternat Med. 2022 Jun 6;2022:8361808. doi: 10.1155/2022/8361808 (PMC9192314; doi:10.1155/2022/8361808)
Supplement: Supplementary Materials — Table S1. The primer sequences used in this present study. Table S2. Information of bioactive compounds in HSYC with good ADME properties. Table S3. Targets of bioactive compounds obtained from databases. Table S4. DOR-related targets. [file 8361808.f1.zip › 8361808.f1/Table S3.pdf]

Table S3 Targets of bioactive compounds obtained from databases

Compound	Target	Source
Isofucosterol	ABAT	symmap
Isofucosterol	ABAT	TCMSP
arachidonic acid	ABCA1	symmap
3'-Methoxydaidzein	ABCB1	ETCM
Isofucosterol	ABCB1	ETCM
Stigmasterol	ABCB1	ETCM
(+)-catechin	ABCB1	ETCM
(+)-catechin	ABCB1	ETCM
3'-Methoxydaidzein	ABCB1	ETCM
3'-Methoxydaidzein	ABCB1	ETCM
sitosterol	ABCB11	ETCM
3'-Methoxydaidzein	ABCC1	ETCM
(+)-catechin	ABCC1	ETCM
(+)-catechin	ABCC1	ETCM
3'-Methoxydaidzein	ABCC1	ETCM
3'-Methoxydaidzein	ABCC1	ETCM
3'-Methoxydaidzein	ABCC2	ETCM
diosgenin	ABCC2	symmap
(+)-catechin	ABCC2	ETCM
3'-Methoxydaidzein	ABCC2	ETCM
arachidonic acid	ABCC4	symmap
arachidonic acid	ABCG1	symmap
arachidonic acid	ABCG1	TCMSP
3'-Methoxydaidzein	ABCG2	ETCM
Isofucosterol	ABCG2	ETCM
quercetin	ABCG2	symmap
Stigmasterol	ABCG2	ETCM
(+)-catechin	ABCG2	ETCM
(+)-catechin	ABCG2	ETCM
3'-Methoxydaidzein	ABCG2	ETCM
3'-Methoxydaidzein	ABCG2	ETCM
3'-Methoxydaidzein	ABCG2	ETCM
sitosterol	ABCG5	ETCM
Stigmasterol	ABCG5	pubchem
sitosterol	ABCG8	ETCM
Stigmasterol	ABCG8	pubchem
quercetin	ACACA	symmap
quercetin	ACACA	TCMSP
sesamin	ACACA	symmap
sesamin	ACACA	TCMSP
sesamin	ACADM	symmap
sesamin	ACADM	TCMSP
Stigmasterol	ACAT1	pubchem
11,14-eicosadienoic acid	ACHE	TCMSP
5Z-eicosenoic acid	ACHE	TCMSP
7-Methoxy-2-methyl isoflavone	ACHE	symmap
7-Methoxy-2-methyl isoflavone	ACHE	TCMSP
arachidonate	ACHE	TCMSP
arachidonic acid	ACHE	TCMSP
beta-sitosterol	ACHE	TCMSP
formononetin	ACHE	symmap
formononetin	ACHE	TCMSP
isorhamnetin	ACHE	symmap
isorhamnetin	ACHE	TCMSP
kaempferol	ACHE	symmap
kaempferol	ACHE	TCMSP
quercetin	ACHE	symmap
quercetin	ACHE	TCMSP
sesamin	ACHE	TCMSP
Stigmasterol	ACHE	TCMSP
Yangambin	ACHE	TCMSP
sesamin	ACLY	symmap
sesamin	ACOX1	symmap
sesamin	ACOX1	TCMSP
quercetin	ACPP	symmap
quercetin	ACPP	TCMSP
3'-Methoxydaidzein	ACTB	ETCM
(+)-catechin	ACTB	ETCM
3'-Methoxydaidzein	ACTB	ETCM
Isofucosterol	ADH1A	TCMSP
Isofucosterol	ADH1B	TCMSP
Isofucosterol	ADH1C	TCMSP
Stigmasterol	ADH1C	TCMSP
beta-sitosterol	ADRA1A	symmap
formononetin	ADRA1A	symmap
7-Methoxy-2-methyl isoflavone	ADRA1B	TCMSP
beta-sitosterol	ADRA1B	TCMSP
kaempferol	ADRA1B	TCMSP
Stigmasterol	ADRA1B	TCMSP
7-Methoxy-2-methyl isoflavone	ADRA1D	TCMSP
beta-sitosterol	ADRA1D	TCMSP
formononetin	ADRA1D	TCMSP
Stigmasterol	ADRA1D	TCMSP
suchilactone	ADRA1D	TCMSP
7-Methoxy-2-methyl isoflavone	ADRB1	symmap
7-Methoxy-2-methyl isoflavone	ADRB1	TCMSP
Stigmasterol	ADRB1	TCMSP
7-Methoxy-2-methyl isoflavone	ADRB2	symmap
7-Methoxy-2-methyl isoflavone	ADRB2	TCMSP
beta-sitosterol	ADRB2	symmap
beta-sitosterol	ADRB2	TCMSP
formononetin	ADRB2	symmap
formononetin	ADRB2	TCMSP
quercetin	ADRB2	symmap
quercetin	ADRB2	TCMSP
Stigmasterol	ADRB2	TCMSP
suchilactone	ADRB2	symmap
suchilactone	ADRB2	TCMSP
3'-Methoxydaidzein	AHR	ETCM
kaempferol	AHR	symmap
kaempferol	AHR	TCMSP
quercetin	AHR	symmap
quercetin	AHR	TCMSP
(+)-catechin	AHR	ETCM
3'-Methoxydaidzein	AHR	ETCM
kaempferol	AHSA1	symmap
quercetin	AHSA1	symmap
isorhamnetin	AKR1B1	symmap
isorhamnetin	AKR1B1	TCMSP
quercetin	AKR1B1	symmap
quercetin	AKR1B1	TCMSP
Stigmasterol	AKR1B1	TCMSP
Isofucosterol	AKR1C1	ETCM
Stigmasterol	AKR1C1	ETCM
Isofucosterol	AKR1C2	ETCM
Stigmasterol	AKR1C2	ETCM
kaempferol	AKR1C3	symmap
kaempferol	AKR1C3	TCMSP
3'-Methoxydaidzein	AKT1	ETCM
diosgenin	AKT1	symmap
kaempferol	AKT1	symmap
quercetin	AKT1	symmap
(+)-catechin	AKT1	ETCM
3'-Methoxydaidzein	AKT1	ETCM
3'-Methoxydaidzein	ALB	ETCM
Isofucosterol	ALB	ETCM
Stigmasterol	ALB	ETCM
(+)-catechin	ALB	ETCM
3'-Methoxydaidzein	ALB	ETCM
arachidonic acid	ALDH2	symmap
arachidonic acid	ALDH2	TCMSP
arachidonic acid	ALDH3A1	symmap
arachidonic acid	ALDH3A1	TCMSP
arachidonic acid	ALOX5	symmap
arachidonic acid	ALOX5	TCMSP
kaempferol	ALOX5	symmap
kaempferol	ALOX5	TCMSP
quercetin	ALOX5	symmap
quercetin	ALOX5	TCMSP
sitosterol	APOE	ETCM
(+)-catechin	AR	TCMSP
(1R,2R,4aS,6aS,6aR,6bR,8aR,12aR	AR	TCMSP
(3S,5R,8R,9R,10S,14S)-3,17-dihyd	AR	TCMSP
3'-Methoxydaidzein	AR	TCMSP
7-Methoxy-2-methyl isoflavone	AR	symmap
7-Methoxy-2-methyl isoflavone	AR	TCMSP
Ammidin	AR	TCMSP
beta-sitosterol	AR	TCMSP
campest-5-en-3beta-ol	AR	TCMSP
CLR	AR	TCMSP
diosgenin	AR	TCMSP
ellagic acid	AR	symmap
ellagic acid	AR	TCMSP
formononetin	AR	symmap
formononetin	AR	TCMSP
Isofucosterol	AR	ETCM
Isofucosterol	AR	TCMSP
isorhamnetin	AR	symmap
isorhamnetin	AR	TCMSP
kaempferol	AR	symmap
kaempferol	AR	TCMSP
Mairin	AR	TCMSP
matrine	AR	symmap
matrine	AR	TCMSP
NSC63551	AR	TCMSP
quercetin	AR	symmap
quercetin	AR	TCMSP
sesamin	AR	TCMSP
sitosterol	AR	TCMSP
Stigmasterol	AR	ETCM
Stigmasterol	AR	TCMSP
suchilactone	AR	TCMSP
Yangambin	AR	TCMSP
3'-Methoxydaidzein	ATP5A1	ETCM
(+)-catechin	ATP5A1	ETCM
3'-Methoxydaidzein	ATP5A1	ETCM
3'-Methoxydaidzein	ATP5B	ETCM
formononetin	ATP5B	symmap
formononetin	ATP5B	TCMSP
(+)-catechin	ATP5B	ETCM
3'-Methoxydaidzein	ATP5B	ETCM
3'-Methoxydaidzein	ATP5C1	ETCM
(+)-catechin	ATP5C1	ETCM
3'-Methoxydaidzein	ATP5C1	ETCM
sesamin	AUH	symmap
kaempferol	BAX	symmap
quercetin	BAX	symmap
sitosterol	BAX	symmap
beta-sitosterol	BCL2	symmap
beta-sitosterol	BCL2	TCMSP
kaempferol	BCL2	symmap
kaempferol	BCL2	TCMSP
quercetin	BCL2	symmap
quercetin	BCL2	TCMSP
sitosterol	BCL2	symmap
quercetin	BCL2L1	symmap
quercetin	BIRC5	symmap
quercetin	BMP2	symmap
arachidonic acid	C1R	symmap
arachidonic acid	C1R	TCMSP
(+)-catechin	CA2	TCMSP
11,14-eicosadienoic acid	CA2	TCMSP
3'-Methoxydaidzein	CA2	TCMSP
7-Methoxy-2-methyl isoflavone	CA2	TCMSP
arachidonate	CA2	TCMSP
beta-sitosterol	CA2	TCMSP
formononetin	CA2	TCMSP
Isofucosterol	CA2	TCMSP
isorhamnetin	CA2	TCMSP
kaempferol	CA2	TCMSP
quercetin	CA2	TCMSP
Stigmasterol	CA2	TCMSP
Yangambin	CA2	TCMSP
(+)-catechin	CALM3	symmap
7-Methoxy-2-methyl isoflavone	CALM3	TCMSP
formononetin	CALM3	TCMSP
isorhamnetin	CALM3	TCMSP
kaempferol	CALM3	TCMSP
Marckine	CALM3	TCMSP
suchilactone	CALM3	TCMSP
Yangambin	CALM3	TCMSP
arachidonic acid	CASP3	symmap
kaempferol	CASP3	symmap
matrine	CASP3	symmap
quercetin	CASP3	symmap
sitosterol	CASP3	ETCM
sitosterol	CASP3	symmap
quercetin	CASP8	symmap
sitosterol	CASP8	symmap
quercetin	CASP9	symmap
sitosterol	CASP9	symmap
(+)-catechin	CAT	symmap
(+)-catechin	CAT	TCMSP
diosgenin	CAT	symmap
diosgenin	CAT	TCMSP
quercetin	CAT	symmap
quercetin	CAV1	symmap
3'-Methoxydaidzein	CBR1	ETCM
(+)-catechin	CBR1	ETCM
3'-Methoxydaidzein	CBR1	ETCM
quercetin	CCL2	symmap
quercetin	CCL2	TCMSP
(+)-catechin	CCNA2	TCMSP
3'-Methoxydaidzein	CCNA2	TCMSP
7-Methoxy-2-methyl isoflavone	CCNA2	symmap
7-Methoxy-2-methyl isoflavone	CCNA2	TCMSP
Ammidin	CCNA2	TCMSP
beta-sitosterol	CCNA2	TCMSP
formononetin	CCNA2	symmap
formononetin	CCNA2	TCMSP
isorhamnetin	CCNA2	symmap
isorhamnetin	CCNA2	TCMSP
kaempferol	CCNA2	TCMSP
Marckine	CCNA2	TCMSP
quercetin	CCNA2	TCMSP
suchilactone	CCNA2	TCMSP
Yangambin	CCNA2	TCMSP
quercetin	CCNB1	symmap
arachidonic acid	CCND1	symmap
quercetin	CCND1	symmap
sesamin	CCND1	symmap
paeoniflorin	CD14	symmap
kaempferol	CD40LG	TCMSP
matrine	CD40LG	symmap
paeoniflorin	CD40LG	TCMSP
quercetin	CD40LG	symmap
quercetin	CD40LG	TCMSP
matrine	CD44	symmap
kaempferol	CDC2	symmap
quercetin	CDC2	symmap
kaempferol	CDK1	TCMSP
quercetin	CDK1	TCMSP
(+)-catechin	CDK2	TCMSP
3'-Methoxydaidzein	CDK2	TCMSP
7-Methoxy-2-methyl isoflavone	CDK2	symmap
7-Methoxy-2-methyl isoflavone	CDK2	TCMSP
Ammidin	CDK2	TCMSP
arachidonic acid	CDK2	symmap
arachidonic acid	CDK2	TCMSP
beta-sitosterol	CDK2	TCMSP
ellagic acid	CDK2	symmap
ellagic acid	CDK2	TCMSP
formononetin	CDK2	symmap
formononetin	CDK2	TCMSP
isorhamnetin	CDK2	symmap
isorhamnetin	CDK2	TCMSP
kaempferol	CDK2	TCMSP
quercetin	CDK2	TCMSP
Stigmasterol	CDK2	TCMSP
suchilactone	CDK2	TCMSP
arachidonic acid	CDK4	symmap
arachidonic acid	CDK4	TCMSP
3'-Methoxydaidzein	CDK6	ETCM
(+)-catechin	CDK6	ETCM
3'-Methoxydaidzein	CDK6	ETCM
diosgenin	CDKN1A	symmap
ellagic acid	CDKN1A	symmap
quercetin	CDKN1A	symmap
quercetin	CDKN2A	symmap
3'-Methoxydaidzein	CEBPB	ETCM
(+)-catechin	CEBPB	ETCM
3'-Methoxydaidzein	CEBPB	ETCM
arachidonic acid	CETP	symmap
(+)-catechin	CHEK1	TCMSP
3'-Methoxydaidzein	CHEK1	TCMSP
7-Methoxy-2-methyl isoflavone	CHEK1	TCMSP
Ammidin	CHEK1	TCMSP
beta-sitosterol	CHEK1	TCMSP
formononetin	CHEK1	TCMSP
isorhamnetin	CHEK1	TCMSP
kaempferol	CHEK1	TCMSP
quercetin	CHEK1	TCMSP
suchilactone	CHEK1	TCMSP
quercetin	CHEK2	symmap
7-Methoxy-2-methyl isoflavone	CHRM1	symmap
7-Methoxy-2-methyl isoflavone	CHRM1	TCMSP
Ammidin	CHRM1	symmap
Ammidin	CHRM1	TCMSP
beta-sitosterol	CHRM1	symmap
beta-sitosterol	CHRM1	TCMSP
formononetin	CHRM1	symmap
formononetin	CHRM1	TCMSP
kaempferol	CHRM1	TCMSP
Stigmasterol	CHRM1	TCMSP
beta-sitosterol	CHRM2	TCMSP
kaempferol	CHRM2	TCMSP
Stigmasterol	CHRM2	TCMSP
7-Methoxy-2-methyl isoflavone	CHRM3	TCMSP
beta-sitosterol	CHRM3	TCMSP
Stigmasterol	CHRM3	TCMSP
beta-sitosterol	CHRM4	TCMSP
7-Methoxy-2-methyl isoflavone	CHRM5	TCMSP
beta-sitosterol	CHRNA2	TCMSP
7-Methoxy-2-methyl isoflavone	CHRNA7	TCMSP
beta-sitosterol	CHRNA7	TCMSP
Stigmasterol	CHRNA7	TCMSP
ellagic acid	CHS2	symmap
quercetin	CHUK	symmap
quercetin	CLDN4	symmap
Isofucosterol	CLEC4E	ETCM
Stigmasterol	CLEC4E	ETCM
quercetin	COL1A1	symmap
quercetin	COL1A1	TCMSP
arachidonic acid	COL1A2	symmap
arachidonic acid	COL1A2	TCMSP
quercetin	COL3A1	symmap
quercetin	COL3A1	TCMSP
3'-Methoxydaidzein	COMT	ETCM
(+)-catechin	COMT	ETCM
3'-Methoxydaidzein	COMT	ETCM
quercetin	CRP	symmap
3'-Methoxydaidzein	CSNK2A1	ETCM
(+)-catechin	CSNK2A1	ETCM
3'-Methoxydaidzein	CSNK2A1	ETCM
3'-Methoxydaidzein	CSNK2B	ETCM
(+)-catechin	CSNK2B	ETCM
3'-Methoxydaidzein	CSNK2B	ETCM
quercetin	CTSD	symmap
quercetin	CTSD	TCMSP
quercetin	CXCL10	symmap
quercetin	CXCL11	symmap
quercetin	CXCL2	symmap
ellagic acid	CXCL8	symmap
quercetin	CXCL8	symmap
Stigmasterol	CXCL8	pubchem
Isofucosterol	CYP11A1	ETCM
Stigmasterol	CYP11A1	ETCM
Isofucosterol	CYP19A1	ETCM
Stigmasterol	CYP19A1	ETCM
kaempferol	CYP1A1	symmap
quercetin	CYP1A1	symmap
Stigmasterol	CYP1A1	ETCM
3'-Methoxydaidzein	CYP1A2	ETCM
kaempferol	CYP1A2	symmap
kaempferol	CYP1A2	TCMSP
quercetin	CYP1A2	symmap
quercetin	CYP1A2	TCMSP
(+)-catechin	CYP1A2	ETCM
3'-Methoxydaidzein	CYP1A2	ETCM
3'-Methoxydaidzein	CYP1A2	ETCM
3'-Methoxydaidzein	CYP1B1	ETCM
Isofucosterol	CYP1B1	ETCM
kaempferol	CYP1B1	symmap
quercetin	CYP1B1	symmap
Stigmasterol	CYP1B1	ETCM
(+)-catechin	CYP1B1	ETCM
(+)-catechin	CYP1B1	ETCM
3'-Methoxydaidzein	CYP1B1	ETCM
3'-Methoxydaidzein	CYP1B1	ETCM
Isofucosterol	CYP24A1	ETCM
Stigmasterol	CYP24A1	ETCM
Isofucosterol	CYP27A1	ETCM
Stigmasterol	CYP27A1	ETCM
Isofucosterol	CYP27B1	ETCM
Stigmasterol	CYP27B1	ETCM
Isofucosterol	CYP2A13	ETCM
Stigmasterol	CYP2A13	ETCM
Isofucosterol	CYP2B6	ETCM
sesamin	CYP2B6	symmap
sesamin	CYP2B6	TCMSP
Stigmasterol	CYP2B6	ETCM
Isofucosterol	CYP2C19	ETCM
Stigmasterol	CYP2C19	ETCM
3'-Methoxydaidzein	CYP2C8	ETCM
Isofucosterol	CYP2C8	ETCM
Stigmasterol	CYP2C8	ETCM
(+)-catechin	CYP2C8	ETCM
3'-Methoxydaidzein	CYP2C8	ETCM
Isofucosterol	CYP2C9	ETCM
Stigmasterol	CYP2C9	ETCM
Isofucosterol	CYP2D6	ETCM
Stigmasterol	CYP2D6	ETCM
Isofucosterol	CYP2J2	ETCM
Stigmasterol	CYP2J2	ETCM
Isofucosterol	CYP2R1	ETCM
Stigmasterol	CYP2R1	ETCM
Isofucosterol	CYP3A4	ETCM
kaempferol	CYP3A4	symmap
kaempferol	CYP3A4	TCMSP
quercetin	CYP3A4	symmap
quercetin	CYP3A4	TCMSP
Stigmasterol	CYP3A4	ETCM
Isofucosterol	CYP3A43	ETCM
Stigmasterol	CYP3A43	ETCM
Isofucosterol	CYP3A5	ETCM
Stigmasterol	CYP3A5	ETCM
Isofucosterol	CYP3A7	ETCM
Stigmasterol	CYP3A7	ETCM
quercetin	DCAF5	symmap
sesamin	DCI	symmap
sesamin	DECR1	symmap
sesamin	DECR1	TCMSP
sitosterol	DHCR24	ETCM
kaempferol	DIO1	symmap
quercetin	DIO1	symmap
(+)-catechin	DPP4	TCMSP
5Z-eicosenoic acid	DPP4	TCMSP
7-Methoxy-2-methyl isoflavone	DPP4	TCMSP
Ammidin	DPP4	TCMSP
arachidonic acid	DPP4	TCMSP
beta-sitosterol	DPP4	TCMSP
CLR	DPP4	TCMSP
formononetin	DPP4	TCMSP
isorhamnetin	DPP4	TCMSP
kaempferol	DPP4	TCMSP
paeoniflorgenone	DPP4	TCMSP
quercetin	DPP4	TCMSP
sesamin	DPP4	TCMSP
Stigmasterol	DPP4	TCMSP
suchilactone	DPP4	TCMSP
Yangambin	DPP4	TCMSP
7-Methoxy-2-methyl isoflavone	DRD1	TCMSP
beta-sitosterol	DRD1	TCMSP
quercetin	DUOX2	symmap
quercetin	E2F1	symmap
quercetin	E2F2	symmap
sesamin	ECE1	symmap
sesamin	ECE1	TCMSP
arachidonic acid	EGF	symmap
arachidonic acid	EGF	TCMSP
quercetin	EGF	symmap
quercetin	EGF	TCMSP
quercetin	EGFR	symmap
quercetin	EGFR	TCMSP
sesamin	EHHADH	symmap
sesamin	EHHADH	TCMSP
3'-Methoxydaidzein	EIF3F	ETCM
(+)-catechin	EIF3F	ETCM
3'-Methoxydaidzein	EIF3F	ETCM
quercetin	EIF6	symmap
quercetin	ELK1	symmap
arachidonic acid	ENSG000001966	TCMSP
quercetin	ERBB2	symmap
quercetin	ERBB3	symmap
(+)-catechin	ESR1	TCMSP
(3S,5R,8R,9R,10S,14S)-3,17-dihyd	ESR1	TCMSP
3'-Methoxydaidzein	ESR1	ETCM
3'-Methoxydaidzein	ESR1	TCMSP
7-Methoxy-2-methyl isoflavone	ESR1	symmap
7-Methoxy-2-methyl isoflavone	ESR1	TCMSP
Ammidin	ESR1	TCMSP
arachidonic acid	ESR1	TCMSP
beta-sitosterol	ESR1	TCMSP
campest-5-en-3beta-ol	ESR1	TCMSP
CLR	ESR1	TCMSP
diosgenin	ESR1	symmap
diosgenin	ESR1	TCMSP
ellagic acid	ESR1	symmap
ellagic acid	ESR1	TCMSP
formononetin	ESR1	symmap
formononetin	ESR1	TCMSP
Isofucosterol	ESR1	ETCM
Isofucosterol	ESR1	TCMSP
isorhamnetin	ESR1	symmap
isorhamnetin	ESR1	TCMSP
kaempferol	ESR1	symmap
kaempferol	ESR1	TCMSP
Mairin	ESR1	TCMSP
Marckine	ESR1	TCMSP
NSC63551	ESR1	TCMSP
quercetin	ESR1	symmap
quercetin	ESR1	TCMSP
sesamin	ESR1	TCMSP
sitosterol	ESR1	TCMSP
Stigmasterol	ESR1	ETCM
Stigmasterol	ESR1	TCMSP
suchilactone	ESR1	TCMSP
Yangambin	ESR1	TCMSP
(+)-catechin	ESR1	ETCM
(+)-catechin	ESR1	ETCM
3'-Methoxydaidzein	ESR1	ETCM
3'-Methoxydaidzein	ESR1	ETCM
(+)-catechin	ESR2	TCMSP
3'-Methoxydaidzein	ESR2	ETCM
3'-Methoxydaidzein	ESR2	TCMSP
7-Methoxy-2-methyl isoflavone	ESR2	symmap
7-Methoxy-2-methyl isoflavone	ESR2	TCMSP
Ammidin	ESR2	TCMSP
beta-sitosterol	ESR2	TCMSP
formononetin	ESR2	symmap
formononetin	ESR2	TCMSP
Isofucosterol	ESR2	ETCM
isorhamnetin	ESR2	symmap
isorhamnetin	ESR2	TCMSP
kaempferol	ESR2	symmap
kaempferol	ESR2	TCMSP
quercetin	ESR2	symmap
quercetin	ESR2	TCMSP
sesamin	ESR2	TCMSP
Stigmasterol	ESR2	ETCM
suchilactone	ESR2	TCMSP
Yangambin	ESR2	TCMSP
(+)-catechin	ESR2	ETCM
(+)-catechin	ESR2	ETCM
3'-Methoxydaidzein	ESR2	ETCM
3'-Methoxydaidzein	ESR2	ETCM
(+)-catechin	ESRRA	ETCM
3'-Methoxydaidzein	ESRRA	ETCM
3'-Methoxydaidzein	ESRRB	ETCM
(+)-catechin	ESRRB	ETCM
3'-Methoxydaidzein	ESRRB	ETCM
quercetin	F10	TCMSP
sesamin	F10	TCMSP
suchilactone	F10	TCMSP
Yangambin	F10	TCMSP
11,14-eicosadienoic acid	F2	TCMSP
5Z-eicosenoic acid	F2	TCMSP
7-Methoxy-2-methyl isoflavone	F2	TCMSP
Ammidin	F2	TCMSP
arachidonate	F2	TCMSP
arachidonic acid	F2	TCMSP
beta-sitosterol	F2	TCMSP
Dihomolinolenic acid	F2	TCMSP
formononetin	F2	TCMSP
isorhamnetin	F2	TCMSP
kaempferol	F2	TCMSP
Marckine	F2	TCMSP
quercetin	F2	TCMSP
Stigmasterol	F2	TCMSP
suchilactone	F2	TCMSP
Yangambin	F2	TCMSP
quercetin	F3	symmap
quercetin	F3	TCMSP
isorhamnetin	F7	TCMSP
kaempferol	F7	TCMSP
quercetin	F7	TCMSP
suchilactone	F7	TCMSP
diosgenin	FASN	symmap
diosgenin	FASN	TCMSP
sesamin	FASN	symmap
sesamin	FASN	TCMSP
quercetin	FOS	symmap
diosgenin	FRAP1	symmap
arachidonic acid	G6PD	symmap
arachidonic acid	G6PD	TCMSP
sesamin	G6PD	symmap
sesamin	G6PD	TCMSP
Isofucosterol	GABRA1	ETCM
Stigmasterol	GABRA1	ETCM
Isofucosterol	GABRA2	ETCM
Stigmasterol	GABRA2	ETCM
Isofucosterol	GABRA3	ETCM
Stigmasterol	GABRA3	ETCM
Isofucosterol	GABRA4	ETCM
Stigmasterol	GABRA4	ETCM
Isofucosterol	GABRA5	ETCM
Stigmasterol	GABRA5	ETCM
Isofucosterol	GABRA6	ETCM
Stigmasterol	GABRA6	ETCM
Isofucosterol	GABRB1	ETCM
Stigmasterol	GABRB1	ETCM
Isofucosterol	GABRB2	ETCM
Stigmasterol	GABRB2	ETCM
Isofucosterol	GABRB3	ETCM
Stigmasterol	GABRB3	ETCM
Isofucosterol	GABRD	ETCM
Stigmasterol	GABRD	ETCM
Isofucosterol	GABRE	ETCM
Stigmasterol	GABRE	ETCM
Isofucosterol	GABRG1	ETCM
Stigmasterol	GABRG1	ETCM
Isofucosterol	GABRG2	ETCM
Stigmasterol	GABRG2	ETCM
Isofucosterol	GABRG3	ETCM
Stigmasterol	GABRG3	ETCM
Isofucosterol	GABRP	ETCM
Stigmasterol	GABRP	ETCM
Isofucosterol	GABRQ	ETCM
Stigmasterol	GABRQ	ETCM
Isofucosterol	GC	ETCM
Stigmasterol	GC	ETCM
quercetin	GJA1	symmap
quercetin	GJA1	TCMSP
arachidonic acid	GLB1	symmap
arachidonic acid	GLB1	TCMSP
3'-Methoxydaidzein	GPER	ETCM
(+)-catechin	GPER	ETCM
3'-Methoxydaidzein	GPER	ETCM
isorhamnetin	GRIA2	symmap
isorhamnetin	GRIA2	TCMSP
Isofucosterol	GRIN1	ETCM
Stigmasterol	GRIN1	ETCM
Isofucosterol	GRIN2A	ETCM
Stigmasterol	GRIN2A	ETCM
Isofucosterol	GRIN2B	ETCM
Stigmasterol	GRIN2B	ETCM
Isofucosterol	GRIN2C	ETCM
Stigmasterol	GRIN2C	ETCM
Isofucosterol	GRIN2D	ETCM
Stigmasterol	GRIN2D	ETCM
Isofucosterol	GRIN3A	ETCM
Stigmasterol	GRIN3A	ETCM
Isofucosterol	GRIN3B	ETCM
Stigmasterol	GRIN3B	ETCM
(+)-catechin	GSK3B	TCMSP
(3S,5R,8R,9R,10S,14S)-3,17-dihyd	GSK3B	pubchem
3'-Methoxydaidzein	GSK3B	TCMSP
7-Methoxy-2-methyl isoflavone	GSK3B	symmap
7-Methoxy-2-methyl isoflavone	GSK3B	TCMSP
Ammidin	GSK3B	TCMSP
beta-sitosterol	GSK3B	TCMSP
formononetin	GSK3B	symmap
formononetin	GSK3B	TCMSP
isorhamnetin	GSK3B	symmap
isorhamnetin	GSK3B	TCMSP
kaempferol	GSK3B	TCMSP
quercetin	GSK3B	symmap
quercetin	GSK3B	TCMSP
sesamin	GSK3B	TCMSP
suchilactone	GSK3B	TCMSP
Yangambin	GSK3B	TCMSP
ellagic acid	GSTA1	symmap
ellagic acid	GSTA1	TCMSP
ellagic acid	GSTA2	symmap
ellagic acid	GSTA2	TCMSP
ellagic acid	GSTM1	symmap
ellagic acid	GSTM1	TCMSP
kaempferol	GSTM1	symmap
kaempferol	GSTM1	TCMSP
quercetin	GSTM1	symmap
quercetin	GSTM1	TCMSP
ellagic acid	GSTM2	symmap
ellagic acid	GSTM2	TCMSP
kaempferol	GSTM2	symmap
kaempferol	GSTM2	TCMSP
quercetin	GSTM2	symmap
quercetin	GSTM2	TCMSP
ellagic acid	GSTP1	symmap
ellagic acid	GSTP1	TCMSP
kaempferol	GSTP1	symmap
kaempferol	GSTP1	TCMSP
quercetin	GSTP1	symmap
quercetin	GSTP1	TCMSP
sesamin	HADHB	symmap
(+)-catechin	HAS2	symmap
kaempferol	HAS2	symmap
quercetin	HAS2	symmap
3'-Methoxydaidzein	HCK	ETCM
(+)-catechin	HCK	ETCM
3'-Methoxydaidzein	HCK	ETCM
arachidonic acid	HERC5	symmap
quercetin	HERC5	symmap
3'-Methoxydaidzein	HIBCH	ETCM
(+)-catechin	HIBCH	ETCM
3'-Methoxydaidzein	HIBCH	ETCM
diosgenin	HIF1A	symmap
quercetin	HIF1A	symmap
quercetin	HK2	symmap
Stigmasterol	HMGCR	pubchem
kaempferol	HMOX1	symmap
kaempferol	HMOX1	TCMSP
quercetin	HMOX1	symmap
quercetin	HMOX1	TCMSP
Isofucosterol	HOXA10	ETCM
matrine	HPSE	symmap
Isofucosterol	HSD17B1	ETCM
Stigmasterol	HSD17B1	ETCM
formononetin	HSD3B1	symmap
formononetin	HSD3B2	symmap
quercetin	HSF1	symmap
(+)-catechin	HSP90AA1	TCMSP
3'-Methoxydaidzein	HSP90AA1	ETCM
3'-Methoxydaidzein	HSP90AA1	TCMSP
7-Methoxy-2-methyl isoflavone	HSP90AA1	TCMSP
beta-sitosterol	HSP90AA1	TCMSP
ellagic acid	HSP90AA1	TCMSP
formononetin	HSP90AA1	symmap
formononetin	HSP90AA1	TCMSP
isorhamnetin	HSP90AA1	TCMSP
kaempferol	HSP90AA1	TCMSP
quercetin	HSP90AA1	TCMSP
suchilactone	HSP90AA1	TCMSP
(+)-catechin	HSP90AA1	ETCM
3'-Methoxydaidzein	HSP90AA1	ETCM
3'-Methoxydaidzein	HSPA2	ETCM
(+)-catechin	HSPA2	ETCM
3'-Methoxydaidzein	HSPA2	ETCM
quercetin	HSPA5	symmap
quercetin	HSPB1	symmap
beta-sitosterol	HTR2A	TCMSP
Stigmasterol	HTR2A	TCMSP
kaempferol	ICAM1	symmap
matrine	ICAM1	symmap
quercetin	ICAM1	symmap
sitosterol	ICAM1	ETCM
matrine	IER3IP1	symmap
quercetin	IFNG	symmap
quercetin	IFNG	TCMSP
ellagic acid	IGF2	symmap
quercetin	IGFBP3	symmap
3'-Methoxydaidzein	IGHG1	ETCM
7-Methoxy-2-methyl isoflavone	IGHG1	ETCM
Isofucosterol	IGHG1	ETCM
Isofucosterol	IGHG2	ETCM
kaempferol	IKBKB	symmap
quercetin	IL10	symmap
sesamin	IL10	symmap
Stigmasterol	IL10	pubchem
quercetin	IL1A	symmap
quercetin	IL1B	symmap
quercetin	IL1B	TCMSP
quercetin	IL2	symmap
quercetin	IL2	TCMSP
formononetin	IL4	symmap
matrine	IL6	symmap
matrine	IL6	TCMSP
paeoniflorin	IL6	symmap
paeoniflorin	IL6	TCMSP
quercetin	IL6	symmap
quercetin	IL6	TCMSP
kaempferol	INSR	symmap
kaempferol	INSR	TCMSP
quercetin	INSR	symmap
quercetin	INSR	TCMSP
quercetin	IRF1	symmap
3'-Methoxydaidzein	JAK1	ETCM
(+)-catechin	JAK1	ETCM
3'-Methoxydaidzein	JAK1	ETCM
beta-sitosterol	JUN	symmap
beta-sitosterol	JUN	TCMSP
formononetin	JUN	symmap
formononetin	JUN	TCMSP
kaempferol	JUN	symmap
kaempferol	JUN	TCMSP
quercetin	JUN	symmap
quercetin	JUN	TCMSP
sitosterol	JUN	TCMSP
beta-sitosterol	KCNH2	symmap
beta-sitosterol	KCNH2	TCMSP
quercetin	KCNH2	symmap
quercetin	KCNH2	TCMSP
suchilactone	KCNH2	symmap
suchilactone	KCNH2	TCMSP
Yangambin	KCNH2	symmap
Yangambin	KCNH2	TCMSP
arachidonic acid	KCNK10	symmap
arachidonic acid	KCNK2	symmap
arachidonic acid	KCNK2	TCMSP
Stigmasterol	KNG1	pubchem
(+)-catechin	LACTBL1	TCMSP
arachidonic acid	LACTBL1	TCMSP
beta-sitosterol	LACTBL1	TCMSP
formononetin	LACTBL1	TCMSP
isorhamnetin	LACTBL1	TCMSP
kaempferol	LACTBL1	TCMSP
paeoniflorgenone	LACTBL1	TCMSP
quercetin	LACTBL1	TCMSP
Stigmasterol	LACTBL1	TCMSP
paeoniflorin	LBP	symmap
Isofucosterol	LSS	ETCM
Stigmasterol	LSS	ETCM
7-Methoxy-2-methyl isoflavone	LTA4H	TCMSP
Stigmasterol	LTA4H	TCMSP
quercetin	LYZ	symmap
Isofucosterol	MAOA	ETCM
Stigmasterol	MAOA	ETCM
7-Methoxy-2-methyl isoflavone	MAOB	symmap
7-Methoxy-2-methyl isoflavone	MAOB	TCMSP
Ammidin	MAOB	symmap
Ammidin	MAOB	TCMSP
formononetin	MAOB	symmap
formononetin	MAOB	TCMSP
isorhamnetin	MAOB	symmap
isorhamnetin	MAOB	TCMSP
quercetin	MAOB	symmap
quercetin	MAOB	TCMSP
Stigmasterol	MAOB	TCMSP
beta-sitosterol	MAP2	symmap
beta-sitosterol	MAP2	TCMSP
sitosterol	MAP2	symmap
arachidonic acid	MAPK1	symmap
arachidonic acid	MAPK1	TCMSP
quercetin	MAPK1	symmap
quercetin	MAPK1	TCMSP
(+)-catechin	MAPK14	TCMSP
3'-Methoxydaidzein	MAPK14	TCMSP
7-Methoxy-2-methyl isoflavone	MAPK14	TCMSP
Ammidin	MAPK14	TCMSP
beta-sitosterol	MAPK14	TCMSP
formononetin	MAPK14	TCMSP
isorhamnetin	MAPK14	TCMSP
kaempferol	MAPK14	TCMSP
quercetin	MAPK14	TCMSP
sesamin	MAPK14	TCMSP
suchilactone	MAPK14	TCMSP
arachidonic acid	MAPK3	symmap
quercetin	MAPK3	symmap
kaempferol	MAPK8	symmap
kaempferol	MAPK8	TCMSP
quercetin	MGAM	symmap
quercetin	MGAM	TCMSP
kaempferol	MMP1	symmap
kaempferol	MMP1	TCMSP
quercetin	MMP1	symmap
quercetin	MMP1	TCMSP
ellagic acid	MMP2	symmap
ellagic acid	MMP2	TCMSP
matrine	MMP2	symmap
matrine	MMP2	TCMSP
quercetin	MMP2	symmap
quercetin	MMP2	TCMSP
quercetin	MMP3	symmap
quercetin	MMP3	TCMSP
ellagic acid	MMP9	symmap
quercetin	MMP9	symmap
quercetin	MPO	symmap
quercetin	MPO	TCMSP
formononetin	MT-ND6	symmap
formononetin	MT-ND6	TCMSP
diosgenin	MTOR	TCMSP
3'-Methoxydaidzein	MTTP	ETCM
3'-Methoxydaidzein	MTTP	ETCM
matrine	MYC	symmap
quercetin	MYC	symmap
isorhamnetin	NCF1	symmap
quercetin	NCF1	symmap
3'-Methoxydaidzein	NCOA1	ETCM
3'-Methoxydaidzein	NCOA1	TCMSP
7-Methoxy-2-methyl isoflavone	NCOA1	symmap
7-Methoxy-2-methyl isoflavone	NCOA1	TCMSP
formononetin	NCOA1	TCMSP
isorhamnetin	NCOA1	TCMSP
Stigmasterol	NCOA1	TCMSP
suchilactone	NCOA1	symmap
suchilactone	NCOA1	TCMSP
(+)-catechin	NCOA1	ETCM
3'-Methoxydaidzein	NCOA1	ETCM
(+)-catechin	NCOA2	TCMSP
11,14-eicosadienoic acid	NCOA2	TCMSP
3'-Methoxydaidzein	NCOA2	ETCM
5Z-eicosenoic acid	NCOA2	TCMSP
7-Methoxy-2-methyl isoflavone	NCOA2	TCMSP
arachidonate	NCOA2	TCMSP
beta-sitosterol	NCOA2	TCMSP
CLR	NCOA2	TCMSP
Isofucosterol	NCOA2	ETCM
Isofucosterol	NCOA2	TCMSP
isorhamnetin	NCOA2	TCMSP
kaempferol	NCOA2	TCMSP
quercetin	NCOA2	TCMSP
sitosterol	NCOA2	TCMSP
Stigmasterol	NCOA2	ETCM
Stigmasterol	NCOA2	TCMSP
Yangambin	NCOA2	TCMSP
(+)-catechin	NCOA2	ETCM
3'-Methoxydaidzein	NCOA2	ETCM
quercetin	NFE2L2	symmap
ellagic acid	NFKBIA	symmap
quercetin	NFKBIA	symmap
quercetin	NKX3-1	symmap
(+)-catechin	NOS2	TCMSP
3'-Methoxydaidzein	NOS2	TCMSP
7-Methoxy-2-methyl isoflavone	NOS2	symmap
7-Methoxy-2-methyl isoflavone	NOS2	TCMSP
Ammidin	NOS2	TCMSP
arachidonate	NOS2	TCMSP
arachidonic acid	NOS2	symmap
arachidonic acid	NOS2	TCMSP
beta-sitosterol	NOS2	TCMSP
formononetin	NOS2	symmap
formononetin	NOS2	TCMSP
isorhamnetin	NOS2	symmap
isorhamnetin	NOS2	TCMSP
kaempferol	NOS2	symmap
kaempferol	NOS2	TCMSP
quercetin	NOS2	symmap
quercetin	NOS2	TCMSP
sesamin	NOS2	symmap
sesamin	NOS2	TCMSP
Stigmasterol	NOS2	TCMSP
suchilactone	NOS2	TCMSP
arachidonic acid	NOS3	symmap
quercetin	NOS3	symmap
sesamin	NOS3	symmap
sesamin	NOX1	symmap
sesamin	NOX3	symmap
Stigmasterol	NPC1L1	pubchem
quercetin	NPEPPS	symmap
quercetin	NQO1	symmap
quercetin	NQO1	TCMSP
3'-Methoxydaidzein	NQO2	ETCM
(+)-catechin	NQO2	ETCM
3'-Methoxydaidzein	NQO2	ETCM
3'-Methoxydaidzein	NR1I2	ETCM
diosgenin	NR1I2	symmap
Isofucosterol	NR1I2	ETCM
kaempferol	NR1I2	symmap
quercetin	NR1I2	symmap
sesamin	NR1I2	symmap
Stigmasterol	NR1I2	ETCM
(+)-catechin	NR1I2	ETCM
(+)-catechin	NR1I2	ETCM
3'-Methoxydaidzein	NR1I2	ETCM
3'-Methoxydaidzein	NR1I2	ETCM
Isofucosterol	NR1I3	ETCM
kaempferol	NR1I3	symmap
quercetin	NR1I3	symmap
Stigmasterol	NR1I3	ETCM
(1R,2R,4aS,6aS,6aR,6bR,8aR,12aR	NR3C1	TCMSP
(3S,5R,8R,9R,10S,14S)-3,17-dihyd	NR3C1	TCMSP
beta-sitosterol	NR3C1	TCMSP
CLR	NR3C1	TCMSP
diosgenin	NR3C1	TCMSP
Isofucosterol	NR3C1	ETCM
Isofucosterol	NR3C1	TCMSP
Mairin	NR3C1	TCMSP
sitosterol	NR3C1	TCMSP
Stigmasterol	NR3C1	ETCM
Stigmasterol	NR3C1	TCMSP
(3S,5R,8R,9R,10S,14S)-3,17-dihyd	NR3C2	TCMSP
(3S,5R,8R,9R,10S,14S)-3,17-dihyd	NR3C2	TCMSP
CLR	NR3C2	symmap
CLR	NR3C2	TCMSP
diosgenin	NR3C2	symmap
diosgenin	NR3C2	TCMSP
Isofucosterol	NR3C2	ETCM
Isofucosterol	NR3C2	symmap
Isofucosterol	NR3C2	TCMSP
sitosterol	NR3C2	symmap
sitosterol	NR3C2	TCMSP
Stigmasterol	NR3C2	ETCM
Stigmasterol	NR3C2	TCMSP
quercetin	ODC1	symmap
quercetin	ODC1	TCMSP
isorhamnetin	OLR1	symmap
7-Methoxy-2-methyl isoflavone	OPRM1	symmap
7-Methoxy-2-methyl isoflavone	OPRM1	TCMSP
beta-sitosterol	OPRM1	symmap
beta-sitosterol	OPRM1	TCMSP
quercetin	PARP1	symmap
quercetin	PCOLCE	symmap
(+)-catechin	PCP4	TCMSP
3'-Methoxydaidzein	PCP4	TCMSP
7-Methoxy-2-methyl isoflavone	PCP4	TCMSP
formononetin	PCP4	TCMSP
isorhamnetin	PCP4	TCMSP
kaempferol	PCP4	TCMSP
Marckine	PCP4	TCMSP
suchilactone	PCP4	TCMSP
Yangambin	PCP4	TCMSP
arachidonic acid	PECAM1	symmap
(3S,5R,8R,9R,10S,14S)-3,17-dihyd	PGR	TCMSP
(3S,5R,8R,9R,10S,14S)-3,17-dihyd	PGR	TCMSP
beta-sitosterol	PGR	TCMSP
beta-sitosterol	PGR	TCMSP
campest-5-en-3beta-ol	PGR	TCMSP
campest-5-en-3beta-ol	PGR	TCMSP
CLR	PGR	symmap
CLR	PGR	TCMSP
diosgenin	PGR	symmap
diosgenin	PGR	TCMSP
ellagic acid	PGR	symmap
ellagic acid	PGR	TCMSP
Isofucosterol	PGR	ETCM
Isofucosterol	PGR	symmap
Isofucosterol	PGR	TCMSP
kaempferol	PGR	symmap
kaempferol	PGR	TCMSP
Mairin	PGR	symmap
Mairin	PGR	TCMSP
NSC63551	PGR	symmap
NSC63551	PGR	TCMSP
sitosterol	PGR	symmap
sitosterol	PGR	TCMSP
Stigmasterol	PGR	ETCM
Stigmasterol	PGR	TCMSP
3'-Methoxydaidzein	PIK3CG	ETCM
(+)-catechin	PIK3CG	ETCM
3'-Methoxydaidzein	PIK3CG	ETCM
3'-Methoxydaidzein	PIM1	ETCM
(+)-catechin	PIM1	ETCM
3'-Methoxydaidzein	PIM1	ETCM
(+)-catechin	PKIA	TCMSP
3'-Methoxydaidzein	PKIA	TCMSP
7-Methoxy-2-methyl isoflavone	PKIA	symmap
7-Methoxy-2-methyl isoflavone	PKIA	TCMSP
Ammidin	PKIA	TCMSP
beta-sitosterol	PKIA	TCMSP
formononetin	PKIA	symmap
formononetin	PKIA	TCMSP
isorhamnetin	PKIA	TCMSP
kaempferol	PKIA	TCMSP
quercetin	PKIA	TCMSP
Stigmasterol	PKIA	TCMSP
suchilactone	PKIA	TCMSP
arachidonic acid	PLA2G4A	symmap
arachidonic acid	PLA2G4A	TCMSP
diosgenin	PLA2G4A	symmap
diosgenin	PLA2G4A	TCMSP
quercetin	PLAT	symmap
quercetin	PLAT	TCMSP
quercetin	PLAU	symmap
quercetin	PLAU	TCMSP
Stigmasterol	PLAU	TCMSP
beta-sitosterol	PON1	symmap
beta-sitosterol	PON1	TCMSP
quercetin	PON1	symmap
quercetin	PON1	TCMSP
sitosterol	PON1	symmap
quercetin	POR	symmap
quercetin	POR	TCMSP
Isofucosterol	PPARA	ETCM
quercetin	PPARA	symmap
Stigmasterol	PPARA	ETCM
isorhamnetin	PPARD	symmap
quercetin	PPARD	symmap
(+)-catechin	PPARG	TCMSP
11,14-eicosadienoic acid	PPARG	TCMSP
3'-Methoxydaidzein	PPARG	TCMSP
5Z-eicosenoic acid	PPARG	TCMSP
7-Methoxy-2-methyl isoflavone	PPARG	TCMSP
Ammidin	PPARG	TCMSP
arachidonate	PPARG	TCMSP
arachidonic acid	PPARG	symmap
arachidonic acid	PPARG	TCMSP
beta-sitosterol	PPARG	TCMSP
Dihomolinolenic acid	PPARG	TCMSP
formononetin	PPARG	symmap
formononetin	PPARG	TCMSP
isorhamnetin	PPARG	TCMSP
kaempferol	PPARG	symmap
kaempferol	PPARG	TCMSP
quercetin	PPARG	symmap
quercetin	PPARG	TCMSP
Stigmasterol	PPARG	TCMSP
suchilactone	PPARG	TCMSP
Yangambin	PPARG	TCMSP
kaempferol	PPP3CA	symmap
arachidonic acid	PPP5C	symmap
quercetin	PRKCA	symmap
sitosterol	PRKCA	symmap
arachidonic acid	PRKCB	symmap
ellagic acid	PRKCB	symmap
quercetin	PRKCB	symmap
3'-Methoxydaidzein	PRSS1	TCMSP
7-Methoxy-2-methyl isoflavone	PRSS1	symmap
7-Methoxy-2-methyl isoflavone	PRSS1	TCMSP
beta-sitosterol	PRSS1	TCMSP
CLR	PRSS1	TCMSP
formononetin	PRSS1	symmap
formononetin	PRSS1	TCMSP
Isofucosterol	PRSS1	TCMSP
isorhamnetin	PRSS1	symmap
isorhamnetin	PRSS1	TCMSP
kaempferol	PRSS1	symmap
kaempferol	PRSS1	TCMSP
quercetin	PRSS1	symmap
quercetin	PRSS1	TCMSP
sesamin	PRSS1	TCMSP
Stigmasterol	PRSS1	TCMSP
suchilactone	PRSS1	TCMSP
Yangambin	PRSS1	TCMSP
kaempferol	PRXC1A	symmap
quercetin	PRXC1A	symmap
quercetin	PSMD3	symmap
quercetin	PSMD3	TCMSP
arachidonic acid	PTEN	symmap
quercetin	PTEN	symmap
quercetin	PTGER3	symmap
quercetin	PTGER3	TCMSP
arachidonic acid	PTGES	symmap
arachidonic acid	PTGES2	symmap
(+)-catechin	PTGS1	symmap
(+)-catechin	PTGS1	TCMSP
3'-Methoxydaidzein	PTGS1	TCMSP
5Z-eicosenoic acid	PTGS1	symmap
5Z-eicosenoic acid	PTGS1	TCMSP
7-Methoxy-2-methyl isoflavone	PTGS1	symmap
7-Methoxy-2-methyl isoflavone	PTGS1	TCMSP
arachidonate	PTGS1	symmap
arachidonate	PTGS1	TCMSP
arachidonic acid	PTGS1	symmap
arachidonic acid	PTGS1	TCMSP
beta-sitosterol	PTGS1	symmap
beta-sitosterol	PTGS1	TCMSP
Dihomolinolenic acid	PTGS1	symmap
Dihomolinolenic acid	PTGS1	TCMSP
formononetin	PTGS1	symmap
formononetin	PTGS1	TCMSP
isorhamnetin	PTGS1	symmap
isorhamnetin	PTGS1	TCMSP
kaempferol	PTGS1	symmap
kaempferol	PTGS1	TCMSP
quercetin	PTGS1	symmap
quercetin	PTGS1	TCMSP
Stigmasterol	PTGS1	TCMSP
suchilactone	PTGS1	symmap
suchilactone	PTGS1	TCMSP
(+)-catechin	PTGS2	symmap
(+)-catechin	PTGS2	TCMSP
3'-Methoxydaidzein	PTGS2	TCMSP
7-Methoxy-2-methyl isoflavone	PTGS2	symmap
7-Methoxy-2-methyl isoflavone	PTGS2	TCMSP
Ammidin	PTGS2	symmap
Ammidin	PTGS2	TCMSP
arachidonate	PTGS2	symmap
arachidonate	PTGS2	TCMSP
arachidonic acid	PTGS2	symmap
arachidonic acid	PTGS2	TCMSP
beta-sitosterol	PTGS2	symmap
beta-sitosterol	PTGS2	TCMSP
Dihomolinolenic acid	PTGS2	symmap
Dihomolinolenic acid	PTGS2	TCMSP
diosgenin	PTGS2	symmap
diosgenin	PTGS2	TCMSP
formononetin	PTGS2	symmap
formononetin	PTGS2	TCMSP
isorhamnetin	PTGS2	symmap
isorhamnetin	PTGS2	TCMSP
kaempferol	PTGS2	symmap
kaempferol	PTGS2	TCMSP
Marckine	PTGS2	symmap
Marckine	PTGS2	TCMSP
quercetin	PTGS2	symmap
quercetin	PTGS2	TCMSP
sesamin	PTGS2	symmap
sesamin	PTGS2	TCMSP
Stigmasterol	PTGS2	TCMSP
suchilactone	PTGS2	symmap
suchilactone	PTGS2	TCMSP
Yangambin	PTGS2	symmap
Yangambin	PTGS2	TCMSP
3'-Methoxydaidzein	PTK2B	ETCM
(+)-catechin	PTK2B	ETCM
3'-Methoxydaidzein	PTK2B	ETCM
(+)-catechin	PTPN1	TCMSP
3'-Methoxydaidzein	PTPN1	TCMSP
7-Methoxy-2-methyl isoflavone	PTPN1	TCMSP
beta-sitosterol	PTPN1	TCMSP
formononetin	PTPN1	TCMSP
isorhamnetin	PTPN1	TCMSP
kaempferol	PTPN1	TCMSP
quercetin	PTPN1	TCMSP
sesamin	PTPN1	TCMSP
suchilactone	PTPN1	TCMSP
isorhamnetin	PYGM	TCMSP
quercetin	RAF1	symmap
quercetin	RASA1	symmap
quercetin	RASSF1	symmap
quercetin	RB1	symmap
quercetin	RB1	TCMSP
arachidonic acid	RELA	symmap
diosgenin	RELA	symmap
ellagic acid	RELA	symmap
isorhamnetin	RELA	symmap
kaempferol	RELA	symmap
matrine	RELA	symmap
quercetin	RELA	symmap
arachidonic acid	RETN	symmap
Isofucosterol	RORA	ETCM
Stigmasterol	RORA	ETCM
quercetin	RUNX1T1	symmap
quercetin	RUNX2	symmap
3'-Methoxydaidzein	RUVBL2	ETCM
(+)-catechin	RUVBL2	ETCM
3'-Methoxydaidzein	RUVBL2	ETCM
(+)-catechin	RXRA	symmap
(+)-catechin	RXRA	TCMSP
3'-Methoxydaidzein	RXRA	TCMSP
7-Methoxy-2-methyl isoflavone	RXRA	symmap
7-Methoxy-2-methyl isoflavone	RXRA	TCMSP
arachidonic acid	RXRA	symmap
arachidonic acid	RXRA	TCMSP
formononetin	RXRA	symmap
formononetin	RXRA	TCMSP
Isofucosterol	RXRA	ETCM
quercetin	RXRA	symmap
quercetin	RXRA	TCMSP
Stigmasterol	RXRA	TCMSP
suchilactone	RXRA	symmap
suchilactone	RXRA	TCMSP
arachidonate	RXRG	TCMSP
arachidonic acid	RXRG	TCMSP
7-Methoxy-2-methyl isoflavone	SCN5A	symmap
7-Methoxy-2-methyl isoflavone	SCN5A	TCMSP
beta-sitosterol	SCN5A	symmap
beta-sitosterol	SCN5A	TCMSP
quercetin	SCN5A	symmap
quercetin	SCN5A	TCMSP
sesamin	SCN5A	symmap
sesamin	SCN5A	TCMSP
Stigmasterol	SCN5A	TCMSP
suchilactone	SCN5A	symmap
suchilactone	SCN5A	TCMSP
Yangambin	SCN5A	symmap
Yangambin	SCN5A	TCMSP
kaempferol	SELE	symmap
kaempferol	SELE	TCMSP
quercetin	SELE	symmap
quercetin	SELE	TCMSP
arachidonic acid	SELP	symmap
arachidonic acid	SELP	TCMSP
Isofucosterol	SERPINA6	ETCM
Stigmasterol	SERPINA6	ETCM
quercetin	SERPINE1	symmap
3'-Methoxydaidzein	SF3B3	ETCM
(+)-catechin	SF3B3	ETCM
3'-Methoxydaidzein	SF3B3	ETCM
3'-Methoxydaidzein	SHBG	ETCM
Isofucosterol	SHBG	ETCM
Stigmasterol	SHBG	ETCM
(+)-catechin	SHBG	ETCM
(+)-catechin	SHBG	ETCM
3'-Methoxydaidzein	SHBG	ETCM
3'-Methoxydaidzein	SHBG	ETCM
3'-Methoxydaidzein	SHBG	ETCM
Isofucosterol	SIGMAR1	ETCM
Stigmasterol	SIGMAR1	ETCM
formononetin	SIRT1	symmap
Isofucosterol	SLC10A1	ETCM
Stigmasterol	SLC10A1	ETCM
Isofucosterol	SLC10A6	ETCM
Stigmasterol	SLC10A6	ETCM
3'-Methoxydaidzein	SLC16A1	ETCM
(+)-catechin	SLC16A1	ETCM
3'-Methoxydaidzein	SLC16A1	ETCM
3'-Methoxydaidzein	SLC16A7	ETCM
(+)-catechin	SLC16A7	ETCM
3'-Methoxydaidzein	SLC16A7	ETCM
Isofucosterol	SLC22A1	ETCM
Stigmasterol	SLC22A1	ETCM
Isofucosterol	SLC22A3	ETCM
Stigmasterol	SLC22A3	ETCM
Isofucosterol	SLC22A4	ETCM
Stigmasterol	SLC22A4	ETCM
Isofucosterol	SLC22A7	ETCM
Stigmasterol	SLC22A7	ETCM
Isofucosterol	SLC22A8	ETCM
Stigmasterol	SLC22A8	ETCM
kaempferol	SLC2A4	symmap
quercetin	SLC2A4	symmap
arachidonic acid	SLC6A2	symmap
arachidonic acid	SLC6A2	TCMSP
kaempferol	SLC6A2	symmap
kaempferol	SLC6A2	TCMSP
Stigmasterol	SLC6A2	TCMSP
7-Methoxy-2-methyl isoflavone	SLC6A3	symmap
7-Methoxy-2-methyl isoflavone	SLC6A3	TCMSP
formononetin	SLC6A3	symmap
formononetin	SLC6A3	TCMSP
Stigmasterol	SLC6A3	TCMSP
7-Methoxy-2-methyl isoflavone	SLC6A4	symmap
7-Methoxy-2-methyl isoflavone	SLC6A4	TCMSP
beta-sitosterol	SLC6A4	symmap
beta-sitosterol	SLC6A4	TCMSP
formononetin	SLC6A4	symmap
formononetin	SLC6A4	TCMSP
Isofucosterol	SLCO1A2	ETCM
Stigmasterol	SLCO1A2	ETCM
Stigmasterol	SLCO1B1	pubchem
3'-Methoxydaidzein	SLCO2B1	ETCM
(+)-catechin	SLCO2B1	ETCM
3'-Methoxydaidzein	SLCO2B1	ETCM
kaempferol	SLPI	symmap
3'-Methoxydaidzein	SOAT1	ETCM
3'-Methoxydaidzein	SOAT1	ETCM
3'-Methoxydaidzein	SOAT2	ETCM
3'-Methoxydaidzein	SOAT2	ETCM
diosgenin	SOD1	symmap
diosgenin	SOD1	TCMSP
quercetin	SOD1	symmap
quercetin	SOD1	TCMSP
quercetin	SOD3	symmap
quercetin	SPP1	symmap
sesamin	SREBF1	symmap
sitosterol	SREBF1	ETCM
sitosterol	SREBF2	ETCM
kaempferol	STAT1	symmap
quercetin	STAT1	symmap
3'-Methoxydaidzein	STK17B	ETCM
(+)-catechin	STK17B	ETCM
3'-Methoxydaidzein	STK17B	ETCM
quercetin	SULT1E1	symmap
quercetin	SULT1E1	TCMSP
Isofucosterol	SULT2A1	ETCM
Stigmasterol	SULT2A1	ETCM
Isofucosterol	SULT2B1	ETCM
Stigmasterol	SULT2B1	ETCM
quercetin	TGFB1	symmap
sitosterol	TGFB1	symmap
quercetin	THBD	symmap
quercetin	THBD	TCMSP
kaempferol	TNF	symmap
matrine	TNF	symmap
paeoniflorin	TNF	symmap
quercetin	TNF	symmap
Stigmasterol	TNF	pubchem
arachidonic acid	TNFRSF1A	symmap
arachidonic acid	TNFRSF1A	TCMSP
arachidonic acid	TNFRSF1B	symmap
arachidonic acid	TNFRSF1B	TCMSP
quercetin	TOP1	symmap
quercetin	TOP1	TCMSP
3'-Methoxydaidzein	TOP2A	ETCM
Daidzein-4,7-diglucoside	TOP2A	TCMSP
kaempferol	TOP2A	TCMSP
quercetin	TOP2A	symmap
quercetin	TOP2A	TCMSP
Yangambin	TOP2A	TCMSP
(+)-catechin	TOP2A	ETCM
3'-Methoxydaidzein	TOP2A	ETCM
diosgenin	P53	symmap
diosgenin	P53	TCMSP
quercetin	P53	symmap
quercetin	P53	TCMSP
arachidonic acid	TRPV1	symmap
3'-Methoxydaidzein	TTR	ETCM
(+)-catechin	TTR	ETCM
3'-Methoxydaidzein	TTR	ETCM
3'-Methoxydaidzein	UBA1	ETCM
(+)-catechin	UBA1	ETCM
3'-Methoxydaidzein	UBA1	ETCM
arachidonic acid	UCP2	symmap
sesamin	UGT1A1	symmap
3'-Methoxydaidzein	UGT3A1	ETCM
(+)-catechin	UGT3A1	ETCM
(+)-catechin	UGT3A1	ETCM
3'-Methoxydaidzein	UGT3A1	ETCM
3'-Methoxydaidzein	UGT3A1	ETCM
kaempferol	VCAM1	symmap
kaempferol	VCAM1	TCMSP
quercetin	VCAM1	symmap
quercetin	VCAM1	TCMSP
Isofucosterol	VDR	ETCM
Stigmasterol	VDR	ETCM
diosgenin	VEGFA	symmap
diosgenin	VEGFA	TCMSP
ellagic acid	VEGFA	symmap
ellagic acid	VEGFA	TCMSP
quercetin	VEGFA	symmap
quercetin	VEGFA	TCMSP
isorhamnetin	XDH	symmap
isorhamnetin	XDH	TCMSP
kaempferol	XDH	symmap
kaempferol	XDH	TCMSP
quercetin	XDH	symmap
quercetin	XDH	TCMSP

(+)-catechin
(1R,2R,4aS,6aS,6aR,6bR,8aR,12aR,14bS)-1,11-dihydroxy-1,2,6a,6b,9,9,12a-heptamethyl-10-oxo-3,4,5,6,6a,7,8,8a,13,14b-decahydro-2H-picene-4a-carboxylic acid
(3S,5R,8R,9R,10S,14S)-3,17-dihydroxy-4,4,8,10,14-pentamethyl-2,3,5,6,7,9-hexahydro-1H-cyclopenta[a]phenanthrene-15,16-dione
11,14-eicosadienoic acid
3'-Methoxydaidzein
5Z-eicosenoic acid
7-Methoxy-2-methyl isoflavone
Ammidin
arachidonate
arachidonic acid
beta-sitosterol
campest-5-en-3beta-ol
CLR
compound
Daidzein-4,7-diglucoside
Dihomolinolenic acid
diosgenin
ellagic acid
formononetin
Isofucosterol
isorhamnetin
kaempferol
Mairin
Marckine
matrine
NSC63551
paeoniflorgenone
paeoniflorin
quercetin
sesamin
sitosterol
Stigmasterol
suchilactone
Yangambin

ABAT
ABCA1
ABCB1
ABCB1
ABCB11
ABCC1
ABCC1
ABCC2
ABCC2
ABCC4
ABCG1
ABCG2
ABCG2
ABCG5
ABCG8
ACACA
ACADM
ACAT1
ACHE
ACLY
ACOX1
ACPP
ACTB
ACTB
ADH1A
ADH1B
ADH1C
ADRA1A
ADRA1B
ADRA1D
ADRB1
ADRB2
AHR
AHR
AHSA1
AKR1B1
AKR1C1
AKR1C2
AKR1C3
AKT1
AKT1
ALB
ALB
ALDH2
ALDH3A1
ALOX5
APOE
AR
ATP5A1
ATP5A1
ATP5B
ATP5B
ATP5C1
ATP5C1
AUH
BAX
BCL2
BCL2L1
BIRC5
BMP2
C1R
CA2
CALM3
CASP3
CASP8
CASP9
CAT
CAV1
CBR1
CBR1
CCL2
CCNA2
CCNB1
CCND1
CD14
CD40LG
CD44
CDC2
CDK1
CDK2
CDK4
CDK6
CDK6
CDKN1A
CDKN2A
CEBPB
CEBPB
CETP
CHEK1
CHEK2
CHRM1
CHRM2
CHRM3
CHRM4
CHRM5
CHRNA2
CHRNA7
CHS2
CHUK
CLDN4
CLEC4E
COL1A1
COL1A2
COL3A1
COMT
COMT
CRP
CSNK2A1
CSNK2A1
CSNK2B
CSNK2B
CTSD
CXCL10
CXCL11
CXCL2
CXCL8
CYP11A1
CYP19A1
CYP1A1
CYP1A2
CYP1A2
CYP1B1
CYP1B1
CYP24A1
CYP27A1
CYP27B1
CYP2A13
CYP2B6
CYP2C19
CYP2C8
CYP2C8
CYP2C9
CYP2D6
CYP2J2
CYP2R1
CYP3A4
CYP3A43
CYP3A5
CYP3A7
DCAF5
DCI
DECR1
DHCR24
DIO1
DPP4
DRD1
DUOX2
E2F1
E2F2
ECE1
EGF
EGFR
EHHADH
EIF3F
EIF3F
EIF6
ELK1
ENSG00000196689
ERBB2
ERBB3
ESR1
ESR1
ESR2
ESR2
ESRRA
ESRRA
ESRRB
ESRRB
F10
F2
F3
F7
FASN
FOS
FRAP1
G6PD
GABRA1
GABRA2
GABRA3
GABRA4
GABRA5
GABRA6
GABRB1
GABRB2
GABRB3
GABRD
GABRE
GABRG1
GABRG2
GABRG3
GABRP
GABRQ
GC
GJA1
GLB1
GPER
GPER
GRIA2
GRIN1
GRIN2A
GRIN2B
GRIN2C
GRIN2D
GRIN3A
GRIN3B
GSK3B
GSTA1
GSTA2
GSTM1
GSTM2
GSTP1
HADHB
HAS2
HCK
HCK
HERC5
HIBCH
HIBCH
HIF1A
HK2
HMGCR
HMOX1
HOXA10
HPSE
HSD17B1
HSD3B1
HSD3B2
HSF1
HSP90AA1
HSP90AA1
HSPA2
HSPA2
HSPA5
HSPB1
HTR2A
ICAM1
IER3IP1
IFNG
IGF2
IGFBP3
IGHG1
IGHG1
IGHG2
IKBKB
IL10
IL1A
IL1B
IL2
IL4
IL6
INSR
IRF1
JAK1
JAK1
JUN
KCNH2
KCNK10
KCNK2
KNG1
LACTBL1
LBP
LSS
LTA4H
LYZ
MAOA
MAOB
MAP2
MAPK1
MAPK14
MAPK3
MAPK8
MGAM
MMP1
MMP2
MMP3
MMP9
MPO
MT-ND6
MTOR
MTTP
MTTP
MYC
NCF1
NCOA1
NCOA1
NCOA2
NCOA2
NFE2L2
NFKBIA
NKX3-1
NOS2
NOS3
NOX1
NOX3
NPC1L1
NPEPPS
NQO1
NQO2
NQO2
NR1I2
NR1I2
NR1I3
NR3C1
NR3C2
ODC1
OLR1
OPRM1
PARP1
PCOLCE
PCP4
PECAM1
PGR
PIK3CG
PIK3CG
PIM1
PIM1
PKIA
PLA2G4A
PLAT
PLAU
PON1
POR
PPARA
PPARD
PPARG
PPP3CA
PPP5C
PRKCA
PRKCB
PRSS1
PRXC1A
PSMD3
PTEN
PTGER3
PTGES
PTGES2
PTGS1
PTGS2
PTK2B
PTK2B
PTPN1
PYGM
RAF1
RASA1
RASSF1
RB1
RELA
RETN
RORA
RUNX1T1
RUNX2
RUVBL2
RUVBL2
RXRA
RXRG
SCN5A
SELE
SELP
SERPINA6
SERPINE1
SF3B3
SF3B3
SHBG
SHBG
SIGMAR1
SIRT1
SLC10A1
SLC10A6
SLC16A1
SLC16A1
SLC16A7
SLC16A7
SLC22A1
SLC22A3
SLC22A4
SLC22A7
SLC22A8
SLC2A4
SLC6A2
SLC6A3
SLC6A4
SLCO1A2
SLCO1B1
SLCO2B1
SLCO2B1
SLPI
SOAT1
SOAT1
SOAT2
SOAT2
SOD1
SOD3
SPP1
SREBF1
SREBF2
STAT1
STK17B
STK17B
SULT1E1
SULT2A1
SULT2B1
TGFB1
THBD
TNF
TNFRSF1A
TNFRSF1B
TOP1
TOP2A
TOP2A
P53
TRPV1
TTR
TTR
UBA1
UBA1
UCP2
UGT1A1
UGT3A1
UGT3A1
VCAM1
VDR
VEGFA
XDH
