# Supplementary material for: Network Pharmacology-Based Prediction and Verification of the Potential Mechanisms of He's Yangchao Formula against Diminished Ovarian Reserve
Source: Evid Based Complement Alternat Med. 2022 Jun 6;2022:8361808. doi: 10.1155/2022/8361808 (PMC9192314; doi:10.1155/2022/8361808)
Supplement: Supplementary Materials — Table S1. The primer sequences used in this present study. Table S2. Information of bioactive compounds in HSYC with good ADME properties. Table S3. Targets of bioactive compounds obtained from databases. Table S4. DOR-related targets. [file 8361808.f1.zip › 8361808.f1/Table S4.pdf]

Table S4 DOR-related
ABO
ADIPOQ
AGRP
AKT1
ALB
ALDH2
AMH
APOE
AR
ARTN
ATG7
BDNF
BMP15
BRCA1
BRCA2
BRD2
CCL2
CCN2
CDH1
CRP
CTNNB1
CXXC5
CYP17A1
CYP19A1
DIAPH2
DICER1
EGFR
EIF4ENIF1
ERBB2
ESR1
ESR2
F9
FASLG
FDPS
FES
FMR1
FOXC1
FOXL2
FOXP3
FSHR
FSTL3
GALT
GATA4
GDF9
GNRH1
GNRHR
GREM1
H19
HRAS
IGF1
IGF2
IGF2R
IL13
IL1B
IL6
INHBB
INS
KRAS
LEP
LGR6
LHCGR
MAPT
MFN2
MIR106A
MIR22
MLH1
MTOR
MYC
NPPB
NR0B1
NR5A1
OPRD1
PALB2
PNPLA2
POF1B
PTEN
PTGS2
RBM12
SERPINF1
SLC2A1
SLCO2A1
SOCS2
SOX9
THBS1
TNF
TNFRSF11B
P53
TP53INP2
TP63
VEGFA
WT1

Gene Syml	Descriptio	Category	Gifts	GC Id	Relevance score	GeneCards Link
BRCA2	BRCA2 DN	Protein Coc	49	GC13P032	93.36	https://www.genecards.org/cgi-bin/carddisp.pl?gene=BRCA2
BRCA1	BRCA1 DN	Protein Coc	50	GC17M043	86.18	https://www.genecards.org/cgi-bin/carddisp.pl?gene=BRCA1
P53	Tumor Prot	Protein Coc	54	GC17M007	55.72	https://www.genecards.org/cgi-bin/carddisp.pl?gene=TP53
CTNNB1	Catenin Be	Protein Coc	53	GC03P041	42.34	https://www.genecards.org/cgi-bin/carddisp.pl?gene=CTNNB1
AKT1	AKT Serin	Protein Coc	54	GC14M104	39.78	https://www.genecards.org/cgi-bin/carddisp.pl?gene=AKT1
ERBB2	Erb-B2 Rec	Protein Coc	54	GC17P039	36.74	https://www.genecards.org/cgi-bin/carddisp.pl?gene=ERBB2
ESR1	Estrogen R	Protein Coc	53	GC06P151	36.65	https://www.genecards.org/cgi-bin/carddisp.pl?gene=ESR1
KRAS	KRAS Prot	Protein Coc	51	GC12M025	35.96	https://www.genecards.org/cgi-bin/carddisp.pl?gene=KRAS
WT1	WT1 Trans	Protein Coc	49	GC11M032	35.91	https://www.genecards.org/cgi-bin/carddisp.pl?gene=WT1
FSHR	Follicle Sti	Protein Coc	49	GC02M048	35.57	https://www.genecards.org/cgi-bin/carddisp.pl?gene=FSHR
NR5A1	Nuclear Re	Protein Coc	50	GC09M124	34.46	https://www.genecards.org/cgi-bin/carddisp.pl?gene=NR5A1
TNF	Tumor Nec	Protein Coc	51	GC06P047	33.79	https://www.genecards.org/cgi-bin/carddisp.pl?gene=TNF
FASLG	Fas Ligand	Protein Coc	47	GC01P172	30.06	https://www.genecards.org/cgi-bin/carddisp.pl?gene=FASLG
MYC	MYC Protc	Protein Coc	51	GC08P127	29.82	https://www.genecards.org/cgi-bin/carddisp.pl?gene=MYC
CDH1	Cadherin 1	Protein Coc	50	GC16P068	28.37	https://www.genecards.org/cgi-bin/carddisp.pl?gene=CDH1
IGF2	Insulin Lik	Protein Coc	48	GC11M002	28.32	https://www.genecards.org/cgi-bin/carddisp.pl?gene=IGF2
DICER1	Dicer 1, Ri	Protein Coc	47	GC14M095	28.04	https://www.genecards.org/cgi-bin/carddisp.pl?gene=DICER1
BMP15	Bone Morp	Protein Coc	41	GC0XP050	27.73	https://www.genecards.org/cgi-bin/carddisp.pl?gene=BMP15
FMR1	FMRP Tra	Protein Coc	44	GC0XP147	27.7	https://www.genecards.org/cgi-bin/carddisp.pl?gene=FMR1
GNRH1	Gonadotro	Protein Coc	41	GC08M025	27.6	https://www.genecards.org/cgi-bin/carddisp.pl?gene=GNRH1
HRAS	HRas Protc	Protein Coc	52	GC11M000	27.44	https://www.genecards.org/cgi-bin/carddisp.pl?gene=HRAS
NR0B1	Nuclear Re	Protein Coc	46	GC0XM03	26.35	https://www.genecards.org/cgi-bin/carddisp.pl?gene=NR0B1
GATA4	GATA Bin	Protein Coc	48	GC08P011	26.02	https://www.genecards.org/cgi-bin/carddisp.pl?gene=GATA4
INS	Insulin	Protein Coc	48	GC11M002	25.96	https://www.genecards.org/cgi-bin/carddisp.pl?gene=INS
FOXL2	Forkhead E	Protein Coc	40	GC03M138	25.92	https://www.genecards.org/cgi-bin/carddisp.pl?gene=FOXL2
CYP19A1	Cytochrom	Protein Coc	48	GC15M051	25.45	https://www.genecards.org/cgi-bin/carddisp.pl?gene=CYP19A1
SOX9	SRY-Box T	Protein Coc	47	GC17P072	24.64	https://www.genecards.org/cgi-bin/carddisp.pl?gene=SOX9
VEGFA	Vascular E	Protein Coc	48	GC06P043	24.39	https://www.genecards.org/cgi-bin/carddisp.pl?gene=VEGFA
IGF1	Insulin Lik	Protein Coc	50	GC12M102	24.2	https://www.genecards.org/cgi-bin/carddisp.pl?gene=IGF1
H19	H19 Imprir	RNA Gene	28	GC11M001	23.81	https://www.genecards.org/cgi-bin/carddisp.pl?gene=H19
SLC2A1	Solute Carr	Protein Coc	52	GC01M042	22.91	https://www.genecards.org/cgi-bin/carddisp.pl?gene=SLC2A1
GDF9	Growth Di	Protein Coc	39	GC05M132	22.87	https://www.genecards.org/cgi-bin/carddisp.pl?gene=GDF9
ESR2	Estrogen R	Protein Coc	49	GC14M064	22.72	https://www.genecards.org/cgi-bin/carddisp.pl?gene=ESR2
EGFR	Epidermal	Protein Coc	54	GC07P055	22.58	https://www.genecards.org/cgi-bin/carddisp.pl?gene=EGFR
MLH1	MutL Hom	Protein Coc	48	GC03P036	22.03	https://www.genecards.org/cgi-bin/carddisp.pl?gene=MLH1
LHCGR	Luteinizing	Protein Coc	47	GC02M048	21.86	https://www.genecards.org/cgi-bin/carddisp.pl?gene=LHCGR
TP63	Tumor Prot	Protein Coc	48	GC03P189	21.59	https://www.genecards.org/cgi-bin/carddisp.pl?gene=TP63
IL6	Interleukin	Protein Coc	50	GC07P022	21.16	https://www.genecards.org/cgi-bin/carddisp.pl?gene=IL6
GNRHR	Gonadotro	Protein Coc	47	GC04M067	21.04	https://www.genecards.org/cgi-bin/carddisp.pl?gene=GNRHR
PTEN	Phosphatas	Protein Coc	52	GC10P087	20.68	https://www.genecards.org/cgi-bin/carddisp.pl?gene=PTEN
PALB2	Partner An	Protein Coc	43	GC16M023	20.61	https://www.genecards.org/cgi-bin/carddisp.pl?gene=PALB2
GALT	Galactose-	Protein Coc	47	GC09P034	20.19	https://www.genecards.org/cgi-bin/carddisp.pl?gene=GALT
AR	Androgen	Protein Coc	53	GC0XP067	20.06	https://www.genecards.org/cgi-bin/carddisp.pl?gene=AR
ALB	Albumin	Protein Coc	50	GC04P073	20.03	https://www.genecards.org/cgi-bin/carddisp.pl?gene=ALB

Disease	Disease_id	Gene	Gene_id	UniProt	ne_Full_Na	rotein_Cla	_diseases_	DSI_g	DPI_g	pLI	Score_gda	EL_gda	EI_gda	N_PMIDs	_SNPs_gd	First_Ref	Last_Ref
Diminished	C3839507	AMH	268	P03971	anti-Mullerian hormon		242	0.476	0.846	3.4E-12	0.1		0.941	17	0	2009	2020
Diminished	C3839507	FMR1	2332	Q06787	FMRP tran	Nucleic aci	346	0.473	0.769	0.64718	0.1		0.857	14	0	2006	2018
Diminished	C3839507	BRD2	6046	P25440	bromodom	Epigenetic	303	0.45	0.808	0.99957	0.06		1	6	0	2011	2019
Diminished	C3839507	OPRD1	4985	P41143	opioid rece	G-protein c	92	0.604	0.731	0.003323	0.03		0.667	3	0	2006	2018
Diminished	C3839507	TP53INP2	58476	Q8IXH6	tumor protein p53 indu		31	0.722	0.385	0.8117	0.03		0.667	3	0	2006	2018
Diminished	C3839507	GDF9	2661	O60383	growth diff	Signaling	44	0.644	0.385	5.52E-06	0.02		1	2	2	2010	2013
Diminished	C3839507	FSHR	2492	P23945	follicle stin	G-protein c	158	0.539	0.654	1.37E-11	0.02		1	2	1	2009	2018
Diminished	C3839507	SLCO2A1	6578	Q92959	solute carri	Transporter	112	0.578	0.654	2.28E-13	0.02		1	2	0	2018	2018
Diminished	C3839507	ESR1	2099	P03372	estrogen re	Nuclear rec	1101	0.324	0.962	0.9992	0.02		1	2	0	2013	2014
Diminished	C3839507	CCN2	1490	P29279	cellular cor	Signaling	518	0.399	0.846	0.000502	0.02		1	2	0	2012	2016
Diminished	C3839507	BRCA1	672	P38398	BRCA1 DN	Enzyme	747	0.367	0.923	9.22E-29	0.01		1	1	0	2015	2015
Diminished	C3839507	FOXP3	50943	Q9BZS1	forkhead box P3		688	0.368	0.846	0.99424	0.01		1	1	0	2017	2017
Diminished	C3839507	CXXC5	51523	Q7LFL8	CXXC finger protein 5		35	0.678	0.423	0.88856	0.01		1	1	0	2012	2012
Diminished	C3839507	SERPINF1	5176	P36955	serpin fami	Enzyme mo	294	0.46	0.846	7.8E-08	0.01		1	1	0	2019	2019
Diminished	C3839507	EIF4ENIF1	56478	Q9NRA8	eukaryotic translation i		14	0.76	0.192	0.99999	0.01		1	1	0	2019	2019
Diminished	C3839507	PNPLA2	57104	Q96AD5	patatin like	Enzyme	199	0.511	0.846	0.004107	0.01		1	1	0	2019	2019
Diminished	C3839507	PTGS2	5743	P35354	prostagland	Enzyme	1234	0.314	0.962	0.99597	0.01		1	1	0	2012	2012
Diminished	C3839507	ARTN	9048	Q5T4W7	artemin	Signaling	248	0.48	0.846	0.35793	0.01		1	1	0	2020	2020
Diminished	C3839507	SOCS2	8835	O14508	suppressor	Enzyme mo	104	0.56	0.731	0.62999	0.01		1	1	0	2012	2012
Diminished	C3839507	LGR6	59352	Q9HBX8	leucine rich	G-protein c	209	0.502	0.923	2.94E-06	0.01		1	1	0	2020	2020
Diminished	C3839507	POF1B	79983	Q8WVV4	POF1B actin binding p		17	0.751	0.308	4.51E-11	0.01		1	1	0	2006	2006
Diminished	C3839507	P53	7157	P04637	tumor prote	Transcripti	2494	0.236	0.962	0.53235	0.01		1	1	1	2020	2020
Diminished	C3839507	CCL2	6347	P13500	C-C motif c	Signaling	1157	0.321	0.962	0.60786	0.01		1	1	0	2017	2017
Diminished	C3839507	MFN2	9927	O95140	mitofusin 2	Enzyme	334	0.471	0.846	0.99415	0.01		1	1	0	2019	2019
Diminished	C3839507	MIR22	407004		microRNA 22		195	0.496	0.808		0.01		1	1	0	2015	2015
Diminished	C3839507	MIR106A	406899		microRNA 106a		140	0.524	0.846		0.01		1	1	0	2018	2018
Diminished	C3839507	FSTL3	10272	O95633	follistatin li	Enzyme mo	33	0.695	0.423	0.68658	0.01		1	1	0	2012	2012
Diminished	C3839507	CRP	1401	P02741	C-reactive protein		1483	0.299	0.962	0.003697	0.01		1	1	0	2017	2017
Diminished	C3839507	DIAPH2	1730	O60879	diaphanous related forı		37	0.678	0.538	0.99443	0.01		1	1	0	2006	2006
Diminished	C3839507	AGRP	181	O00253	agouti relat	Signaling	233	0.488	0.769	0.000699	0.01		1	1	0	2020	2020
Diminished	C3839507	F9	2158	P00740	coagulation	Enzyme	276	0.465	0.885	0.99776	0.01		1	1	0	2012	2012
Diminished	C3839507	FDPS	2224	P14324	farnesyl dip	Enzyme	83	0.599	0.769	0.00562	0.01		1	1	0	2018	2018
Diminished	C3839507	FES	2242	P07332	FES proto-	Kinase	101	0.593	0.769	3.79E-15	0.01		1	1	0	2018	2018
Diminished	C3839507	FOXC1	2296	Q12948	forkhead bo	Transcripti	242	0.483	0.846	0.95337	0.01		1	1	0	2012	2012
Diminished	C3839507	NR5A1	2516	Q13285	nuclear rec	Nuclear rec	255	0.479	0.731	0.9896	0.01		1	1	0	2019	2019
Diminished	C3839507	GREM1	26585	O60565	gremlin 1, DAN family		179	0.508	0.808	0.47436	0.01		1	1	0	2012	2012
Diminished	C3839507	ABO	28	P16442	ABO, alpha	Enzyme	443	0.427	0.885		0.01		1	1	0	2014	2014
Diminished	C3839507	IGF1	3479	P05019	insulin like growth fact		1206	0.318	0.885	0.2716	0.01		1	1	0	2011	2011
Diminished	C3839507	IGF2	3481	P01344	insulin like growth fact		604	0.39	0.885	0.044127	0.01		1	1	0	2011	2011
Diminished	C3839507	IGF2R	3482	P11717	insulin like	Receptor	166	0.518	0.731	1	0.01		1	1	0	2011	2011
Diminished	C3839507	AR	367	P10275	androgen re	Nuclear rec	854	0.351	0.846	0.98837	0.01		1	1	0	2017	2017
Diminished	C3839507	LHCGR	3973	P22888	luteinizing	G-protein c	151	0.528	0.692	5.3E-08	0.01		1	1	0	2012	2012
Diminished	C3839507	RBM12	10137	Q9NTZ6	RNA bindi	Nucleic aci	17	0.769	0.269	0.002109	0.01		1	1	0	2017	2017

tax_id	Org_name	GeneID	CurrentID	Status	Symbol	Aliases	descriptior	other_desi	map_locati	chromosom	genomic_n	start_positio	end_position	orientatior	exon_coun	OMIM
9606	Homo sapi	672	0	live	BRCA1	BRCAI, BI	BRCA1 D	breast canc	17q21.31	17	NC_00001	43044295	43125364	minus	24	113705
9606	Homo sapi	4609	0	live	MYC	MRTLC, b	MYC proto	myc proto-	8q24.21	8	NC_00000	127735434	127742951	plus	3	190080
9606	Homo sapi	2332	0	live	FMR1	FMRP, FR	FMRP tran	synaptic fu	Xq27.3	X	NC_00002	147911919	147951125	plus	17	309550
9606	Homo sapi	7124	0	live	TNF	DIF-alpha,	tumor necr	tumor necr	6p21.33	6	NC_00000	31575565	31578336	plus	4	191160
9606	Homo sapi	2099	0	live	ESR1	ER, ESR, E	estrogen re	estrogen re	6q25.1-q25	6	NC_00000	151654148	152129619	plus	23	133430
9606	Homo sapi	3569	0	live	IL6	BSF-2, BS	interleukin	interleukin-	7p15.3	7	NC_00000	22725889	22732002	plus	6	147620
9606	Homo sapi	268	0	live	AMH	MIF, MIS	anti-Muller	muellerian-	19p13.3	19	NC_00001	2249323	2252073	plus	5	600957
9606	Homo sapi	3952	0	live	LEP	LEPD, OB,	leptin	leptin|leptir	7q32.1	7	NC_00000	128241201	128257629	plus	3	164160
9606	Homo sapi	367	0	live	AR	AIS8, DHT	androgen r	androgen re	Xq12	X	NC_00002	67544021	67730619	plus	11	313700
9606	Homo sapi	9370	0	live	ADIPOQ	ACDC, AC	adiponectir	adiponectir	3q27.3	3	NC_00000	186842710	186858463	plus	4	605441
9606	Homo sapi	348	0	live	APOE	AD2, APO	apolipoprot	apolipoprot	19q13.32	19	NC_00001	44905796	44909393	plus	6	107741
9606	Homo sapi	2475	0	live	MTOR	FRAP, FR	mechanistic	serine/threc	1p36.22	1	NC_00000	11106535	11273497	minus	60	601231
9606	Homo sapi	3479	0	live	IGF1	IGF, IGF-I,	insulin like	insulin-like	12q23.2	12	NC_00001	102395860	102481839	minus	7	147440
9606	Homo sapi	2516	0	live	NR5A1	AD4BP, EI	nuclear rec	steroidoger	9q33.3	9	NC_00000	124481236	124507399	minus	7	184757
9606	Homo sapi	627	0	live	BDNF	ANON2, B	brain deriv	brain-deriv	11p14.1	11	NC_00001	27654893	27722030	minus	12	113505
9606	Homo sapi	3553	0	live	IL1B	IL-1, IL1-E	interleukin	interleukin-	2q14.1	2	NC_00000	112829751	112836843	minus	7	147720
9606	Homo sapi	2661	0	live	GDF9	POF14	growth diff	growth/diff	5q31.1	5	NC_00000	132861181	132866884	minus	6	601918
9606	Homo sapi	4137	0	live	MAPT	DDPAC, F	microtubul	microtubul	17q21.31	17	NC_00001	45894538	46028334	plus	16	157140
9606	Homo sapi	4879	0	live	NPPB	BNP	natriuretic	natriuretic	1p36.22	1	NC_00000	11857464	11858945	minus	3	600295
9606	Homo sapi	7057	0	live	THBS1	THBS, TH	thrombosp	thrombosp	15q14	15	NC_00001	39581079	39599466	plus	22	188060
9606	Homo sapi	1586	0	live	CYP17A1	CPT7, CYI	cytochrom	steroid 17-a	10q24.32	10	NC_00001	102830531	102837413	minus	8	609300
9606	Homo sapi	4982	0	live	TNFRSF11	OCIF, OPC	TNF recept	tumor necr	8q24.12	8	NC_00000	118923557	118951885	minus	5	602643
9606	Homo sapi	217	0	live	ALDH2	ALDH-E2,	aldehyde d	aldehyde d	12q24.12	12	NC_00001	111766933	111817532	plus	13	100650
9606	Homo sapi	3596	0	live	IL13	IL-13, P60	interleukin	interleukin-	5q31.1	5	NC_00000	132656522	132661110	plus	6	147683
9606	Homo sapi	3625	0	live	INHBB		inhibin sub	inhibin beta	2q14.2	2	NC_00000	120346136	120351803	plus	2	147390
9606	Homo sapi	407004	0	live	MIR22	MIRN22, h	microRNA 22		17p13.3	17	NC_00001	1713903	1713987	minus	1	612077
9606	Homo sapi	406899	0	live	MIR106A	MIRN106A	microRNA	hsa-mir-10	Xq26.2	X	NC_00002	134170198	134170278	minus	1	300792
9606	Homo sapi	10533	0	live	ATG7	APG7-LIK	autophagy	ubiquitin-li	3p25.3	3	NC_00000	11272348	11564652	plus	29	608760
